# Hypoxia disrupt tight junctions and promote metastasis of oral squamous cell carcinoma via loss of par3

**DOI:** 10.1186/s12935-023-02924-8

**Published:** 2023-04-24

**Authors:** Shihyun Kim, Suyeon Park, Eun-Hye Moon, Gi Jin Kim, Jongho Choi

**Affiliations:** 1 Department of Oral Pathology, College of Dentistry, Gangneung-Wonju National University, 7 Jukheon-gil, Gangneung- si, Gangwon-do 25457 Republic of Korea; 2 Institute of Lee Gil Ya Cancer and Diabetes, Gachon University, Incheon, 21999 Republic of Korea; 3 Department of Biomedical Science, CHA University, Seongnam, Gyeonggi-do 13488 Republic of Korea

**Keywords:** Oral squamous cell carcinoma, Hypoxia, HIF-1α, TJs, Par3

## Abstract

**Background:**

Oral squamous cell carcinoma (OSCC) is a highly malignant tumor that is frequently associated with lymph node metastasis, resulting in poor prognosis and survival in patients. In the tumor microenvironment, hypoxia plays an important role in regulating cellular responses such as progressive and rapid growth and metastasis. In these processes, tumor cells autonomously undergo diverse transitions and acquire functions. However, hypoxia-induced transition of OSCC and the involvement of hypoxia in OSCC metastasis remain unclear. Therefore, in this study, we aimed to elucidate the mechanism of hypoxia-induced OSCC metastasis and particularly, its impact on tight junctions (TJs).

**Methods:**

The expression of hypoxia-inducible factor 1-alpha (HIF-1α) was detected in tumor tissues and adjacent normal tissues from 29 patients with OSCC using reverse transcription quantitative real-time polymerase chain reaction (qRT-PCR), western blotting, and immunohistochemistry (IHC). The migration and invasion abilities of OSCC cell lines treated with small interfering (si)RNA targeting HIF-1α or cultured in hypoxic conditions were analyzed using Transwell assays. The effect of HIF-1α expression on in vivo tumor metastasis of OSCC cells was evaluated using lung metastasis model.

**Results:**

HIF-1α was overexpressed in patients with OSCC. OSCC metastasis was correlated with HIF-1α expression in OSCC tissues. Hypoxia increased the migration and invasion abilities of OSCC cell lines by regulating the expression and localization of partitioning-defective protein 3 (Par3) and TJs. Furthermore, HIF-1α silencing effectively decreased the invasion and migration abilities of OSCC cell lines and restored TJ expression and localization via Par3. The expression of HIF-1α was positively regulated the OSCC metastasis in vivo.

**Conclusions:**

Hypoxia promotes OSCC metastasis by regulating the expression and localization of Par3 and TJ proteins. HIF-1α positively correlates to OSCC metastasis. Lastly, HIF-1α expression could regulate the expression of Par3 and TJs in OSCC. This finding may aid in elucidating the molecular mechanisms of OSCC metastasis and progression and developing new diagnostic and therapeutic approaches for OSCC metastasis.

**Supplementary Information:**

The online version contains supplementary material available at 10.1186/s12935-023-02924-8.

## Background

Oral squamous cell carcinoma (OSCC) is an epithelial neoplasm of the oral cavity and the one of the 10 most prevalent malignant tumors worldwide [1, 2]. Despite clinical advances in detection, surgery, and treatment, the five-year survival rate of patients with OSCC remains below 42%, which is mainly because of local recurrence and distant metastasis in the first two years of disease due to a lack of specific diagnostic and treatment methods for OSCC metastasis [3, 4]. Furthermore, patients with metastatic tumors must have their neck lymph nodes excised, resulting in postoperative dysfunction and maxillofacial deformities [5, 6]. To facilitate the early diagnosis and development of effective therapeutic approaches for OSCC, it is necessary to identify biological markers associated with OSCC metastasis and elucidate the underlying molecular mechanisms.

Hypoxia is an important feature of the tumor microenvironment in most solid tumors. It results from excessive oxygen consumption by the growing tumor and is associated with local tumor progression and tumor invasiveness, treatment failure, and poor survival [7, 8]. Hypoxia has various effects on tumor cells, and recently, it was reported that hypoxia affects tumors by centrally mediating the expression of the hypoxia-inducible factor (HIF) family members [9, 10]. Hypoxia-induced cellular adaptation, mediated mainly by hypoxia-inducible factors (HIFs), is associated with changes in cell proliferation and metabolism, phenotype conversion, and metastasis [11–13]. HIF-1α, as the major mediator of the response to hypoxia, transcriptionally activates genes in response to hypoxia [14]. It is highly expressed in tumors and involved in tumor stage and aggressive phenotype in various malignant cancers, including liver [15], lung [16], and breast cancers [17]. In head and neck cancer (HNSC), hypoxia-induced HIF-1α overexpression promotes tumor migration and invasion via morphologic transition, matrix metalloproteinase (MMP) activity, and nuclear deformation [18]. The morphologic transition of epithelial cells to a mesenchymal phenotype induced by hypoxic conditions, termed epithelial-to-mesenchymal transition (EMT), causes the primary tumor to switch to a metastatic and invasive type [19, 20]. However, the functional mechanism underlying hypoxia-driven OSCC metastasis remains unclear.

In epithelial cells, adherens junctions (AJs) and tight junctions (TJs), which are structures anchored to the cellular membrane, determine the morphologic homeostasis by establishing cell–cell adhesion [21]. Most cancers including OSCC originate from epithelial cells, and the two junctions undergo dynamic changes in the TME during tumor metastasis [22]. Recently, it was reported that the expression of the AJ protein E-cadherin, which negatively serves as a tumor suppressor, is lost in circulating tumor cells during metastasis to the lung and is associated with EMT [23]. E-Cadherin, a major factor in the AJs, regulates tumor growth and metastasis, and the loss of its expression is a hallmark of the initial step of EMT. E-Cadherin is known to regulate the expression of Snail and Slug, which are markers of mesenchymal transition [24]. However, the mechanisms by which E-cadherin regulates the TME and the role of TJs in OSCC growth and metastasis are largely unknown.

Recent evidence suggest that dissociation of the Par complex of Par3, Par6, and aPKC is essential for the disassembly of AJs and TJs and EMT [25, 26]. Par3, a single scaffold protein, regulates and binds to other Par family members via PDZ, N-terminal, and C-terminal domains. The PDZ domain of Par3 binds to several proteins, such as Par6 and phosphatase and tension homologue [27, 28]. The C-terminal domain of Par3 is involved in the delivery of aPKC to the apical boundary [29]. Par3 suppresses tumor invasion, and downregulation and dissociation of Par3 are associated with a higher tumor grade and invasive phenotype [30–32]. However, studies indicating a functional role of Par3 in OSCC metastasis are very limited, and the molecular mechanisms of Par3 in OSCC remain unclear.

We aimed to determine the potential role of hypoxia in OSCC tumorigenesis and metastasis. First, we investigated whether OSCC migration and invasion depend on hypoxia-induced HIF-1α expression in three OSCC cell lines (HSC-2, SCC-9, and SCC-25). Furthermore, we investigated the changes in the expression of Par3 and junction proteins and correlations between them in these OSCC cell lines under hypoxic conditions.

## Materials and methods

### Clinical specimen and human tissue samples

For expression analysis in HNSC and OSCC, we obtained the clinical information and mRNA expression from The Cancer Genome Atlas (TCGA) data base (http://gdc.cancer.gov) or the Gene expression omnibus (GEO, http://www.ncbi.nlm.nih.gov/geo/). The raw data were manually downloaded from website, and the HIF-1α or Par3 related data was converted to a log2 scale.

The study was approved by the Research Ethics Review Committee of Gangneung-Wonju National University (IRB No. GWNUIRB-2020-26-1). Paired tumor and adjacent normal tissue specimens were obtained from 29 patients with OSCC at Gangneung-Wonju Dental Hospital National University, Inje Paik University, Pusan National University, and Keimyung University Dongsan Hospital (a member of the Korea Biobank Network). All participants underwent surgery, and OSCC diagnosis was determined histopathologically by a pathologist using tissue fragments from the oral region. The clinicopathological features of the study cohort are listed in Additional file 1: Table S1.

### Cell culture

The HSC-2, SCC-9, and SCC-25 OSCC cell lines were obtained from the Japanese Collection of Research Bioresources Cell Bank (Ibaraki, Osaka, Japan) and the American Type Culture Collection (Manassas, VA, USA). HSC-2 cells were cultured in high-glucose Dulbecco’s modified Eagle’s medium (DMEM) (Invitrogen, Carlsbad, CA, USA) supplemented with 1% penicillin/streptomycin and 10% fetal bovine serum (FBS; Gibco, Waltham, MA, USA). SCC-9 and SCC-25 cells were cultured in DMEM/F-12 (Gibco) supplemented with 1% penicillin/streptomycin and 10% FBS. Cultures were incubated at 37 °C under normoxic conditions in a humidified atmosphere of 5% CO_2_ or under hypoxic conditions with 5% CO_2_ and 1.5% O_2_ balanced with N_2_ in a Heracell™ 150i CO_2_ incubator (Thermo Fisher Scientific, Waltham, MA, USA).

Transient transfections of HSC-2, SCC-9, and SCC-25 cells with siRNA (Invitrogen) or negative control siRNA (Santa Cruz Biotechnology, Santa Cruz, CA, USA) were performed using Lipofectamine 2000 (Invitrogen) according to the manufacturer’s protocol. Briefly, cells were cultured in reduced-serum Opti-MEM (Gibco) until they reached 60% confluence. Then, the cells were transfected with 50 nM of siRNA-HIF-1α or siRNA-Par3 at 37 °C under normoxic or hypoxic conditions as mentioned above for 24 h. The siRNA sequences were as follows: siRNA-HIF-1α: 5′-CAU GAA AGC ACA GAU GAA-3′; siRNA-Par3: 5′-AGA CUC UCA CUC AAG ACU-3′.

### Proximity ligation assay (PLA)

The close proximity between Par3 and TJ was investigated by a PLA using the Duolink In situ Red Starter Kit Mouse/Rabbit (Millipore, St. Louis, MO, USA) according to the manufacturer’s protocol. SCC-25 cells at a density of 3 × 10^5^ were cultured on coverslips with or without siRNA-HIF-1α or siRNA-Par3 at 37 °C under normoxic or hypoxic conditions, and then fixed and permeabilized with 4% paraformaldehyde (PFA) and phosphate-buffered saline (PBS) with 0.01% Tween-20 (PBS-T) at room temperature for 15 min. The cells were blocked with blocking solution for 1 h and then incubated with anti-Par3 and anti-TJ1 primary antibodies in 3% bovine serum albumin (BSA; Sigma) in PBS at 4 °C overnight, followed by ligation and amplification with anti-rabbit and anti-mouse PLA probes in the provided buffers. Finally, the cells were mounted with Duolink^®^ In Situ Mounting Medium with 4′,6-diamidino-2-phenylindole (DAPI). Images were acquired using a confocal microscope (Leica Microsystems, Wetzlar, Germany) with Z-stack. Image acquisition settings of the confocal microscope for all images were the same. The PLA signal was captured at a resolution of at least 5 pixels, and the total dots per cell (cells identified by DAPI nuclear stain) were determined using ImageJ software (National Institutes of Health, Bethesda, MD, USA).

### Transwell migration and invasion assays

Migration and invasion assays were performed in 24-well cell culture plates with 8.0-µm-pore Transwell inserts (Corning, Inc., Corning, NY, USA). For the invasion assay, the membranes were coated with Matrigel (BD Biosciences, San Jose, CA, USA), and the subsequent steps were the same as those in the migration assay. HSC-2 cells (8 × 10^3^) and SCC-9 cells (4 × 10^4^) were transfected with siRNA-HIF-1α or siRNA-Par3 and cultured in inserts in Opti-MEM (Gibco) at 37 °C in the presence of 5% CO_2_ overnight. The next day, Opti-MEM was added to the upper chamber and 10% FBS medium to the lower chamber, and the plates were incubated for 24 h. The inserts were fixed and stained with Mayer’s hematoxylin histological staining reagent (Dako, Santa Clara, CA, USA). After washing the inserts with PBS, migrated and invaded cells were counted under an inverted light microscope using an Olympus U-TV camera (Olympus, Tokyo, Japan). Migrated or invaded cells were counted in eight random fields. All assays were performed in triplicate.

### Gelatin zymography

To detect MMP-2 and MMP-9 activities in OSCC cells, supernatants were harvested during an invasion assay conducted as described above. Fifteen microliters of supernatants were mixed with 5 µL of sample buffer (0.125 M Tris-HCL containing 4% sodium dodecyl sulfate (SDS), 0.01% bromophenol, 20% glycerol, pH 6.8) and incubated at room temperature for 20 min. The mixtures were loaded onto a 12% SDS polyacrylamide gel containing 1% gelatin (Sigma-Aldrich, St. Louis, MO, USA). After separation, the gels were washed with 1X renaturation buffer (Bio-Rad Laboratories, Hercules, CA, USA) for 15 min and then incubated with 1X development buffer (Bio-Rad) at 37 °C for 24 h. After development, the gels were stained with a fixation solution containing 10% acetic acid, 20% methanol, and 0.5% Coomassie Brilliant Blue G-250 (Sigma-Aldrich). To obtain a clear band, the gels were rinsed with the fixation solution at room temperature for 3 h. MMP activity was detected as an unstained band and was quantified using the ImageJ software.

### Cell proliferation assay

Cell proliferation was assayed using the Click-iT™ 5-ethynyl-2′-deoxyuridine (EdU) Cell Proliferation Kit (Abcam, Waltham, MA, USA) according to the manufacturer’s protocol. Briefly, HSC-2 and SCC-9 cells at a density of 2 × 10^5^ were seeded on coverslips in 6-well plates and incubated with 10 µM EdU at 37 °C for 2 h. Then, the cells were washed with PBS, fixed with 4% PFA, and permeabilized with 0.5% Triton X-100 for 15 min. Following a brief wash with PBS-T, the click reaction cocktail provided in the Click-iT™ kit was added to the coverslips, which were then incubated in the dark at room temperature for 30 min. After washing with PBS-T, the samples were stained with DAPI for 10 min. Images were captured under a fluorescence microscope using an Olympus U-TV camera, and EdU-labeled cells were counted using the ImageJ software.

### Reverse transcription quantitative real-time polymerase chain reaction (qRT-PCR)

qRT-PCR was used to assess mRNA expression in human and mouse tissues. Total RNA was isolated from human and mouse tissues using TRIzol reagent (Invitrogen). cDNA (500 ng) was synthesized using AccuPower® RocketScript™ Cycle RT PreMix (Bioneer, Daejeon, Korea) according to the manufacturer’s protocol. qPCRs were run on a CFX96 Touch Real-Time PCR system (Bio-Rad) using SYBR Green Master Mix (Bioneer), as described previously [33]. The relative target gene expression levels were calculated using the 2^–ΔΔCT^ method, and the mRNA expression level of GAPDH was used for normalization. The sequences of the human gene-specific primers were as follows: HIF-1α forward 5′-CAU GAA AGC ACA GAU GAA U-3′ and reverse 5′-AUU CAU CUG UGC UUU CAU G-3′; PAR3 forward 5′-CGG TCA AAA GAG AAC CAC GCA G-3′ and reverse 5′-CAT TCA CCC GAA GCC TTC CAT C-3′; TJ protein 1 (TJP1) forward 5′-GTC CAG AAT CTC GGA AAA GTG CC-3′ and reverse 5′-CTT TCA GCG CAC CAT ACC AAC C-3′; and GAPDH forward 5′-CAA AGT TGT CAT GGA TGA CC-3′ and reverse 5′-CCA TGG AGA AGG CTG GGG-3′. Additionally, the sequences of the mouse gene-specific primers for in vivo analysis were as follows: HIF-1α forward 5′-CCT GCA CTG AAT CAA GAG GTT GC-3′ and reverse 5′-CCA TCA GAA GGA CTT GCT GGC T-3′; Par3 forward 5′-GAG ACT CTA CGG AGG TCC ATG T-3′ and reverse 5′-TCG GTC ATC CAG TTC TGT CTC G-3′; TJP1 forward 5′-GTT GGT ACG GTG CCC TGA AAG A-3′ and reverse 5′-GCT GAC AGG TAG GAC AGA CGA T-3′; and GAPDH forward 5′-CAT CAC TGC CAC CCA GAA GAC TG-3′ and reverse 5′-ATG CCA GTG AGC TTC CCG TTC AG-3′. All assays were conducted in triplicates.

### Western blotting

For transfection, HSC-2, SCC-9, and SCC-25 cells at a density of 5 × 10^5^ were seeded in 60 mm cell culture dishes with reduced-serum Opti-MEM (Gibco) and cultured until they reached 60% confluence. And the cells were then transfected with 50 nM siRNA-HIF-1α or siRNA-Par3 at 37 °C under normoxic or hypoxic conditions as mentioned above for 24 h. Total protein was extracted from OSCC cell lines and tissues using 1× RIPA buffer (Sigma) containing a protease inhibitor cocktail (Roche Diagnostics, Basel, Switzerland) and pooled. Aliquots of 50 µg of protein were loaded onto 8–15% SDS polyacrylamide gels, electrophoresed, and transferred to nitrocellulose membranes. The membranes were blocked with 5% BSA in PBS-T at room temperature for 40 min and incubated with primary antibodies at 4 °C overnight. The following antibodies were used: mouse anti-E-cadherin (Cell Signaling Technology, Danvers, MA, USA, 1:1,000 dilution), anti-Par3 (R&D System, Minneapolis, MN, USA, 1:1,000 dilution), anti-p53 (Cell Signaling Technology, 1:1,000 dilution), anti-Par6b and anti-aPKC (Santa Cruz, 1:1,000 dilution), rabbit anti-Snail (Cell Signaling Technology, 1:1,000 dilution), anti-TJP1 (Novus Biologicals, Centennial, CO, USA, 1:1,000 dilution), anti-claudin-1 (Cell Signaling Technology, 1:1,000 dilution), anti-HIF-1α (GeneTex, Inc., Irvine, CA, USA, 1:1,000 dilution), and anti-GAPDH (AB Frontier, Seoul, Republic of Korea, 1:3,000 dilution). After washing with PBS-T, the membranes were blocked with horseradish peroxidase-conjugated anti-mouse and anti-rabbit IgG (Cell Signaling, 1:5,000 dilution) at room temperature for 1 h. Proteins were detected using a chemiluminescence reagent (Millipore) and the FUSION Solo S imaging system (Vilber, Eberhardzell, Germany). Band intensities were quantified using ImageJ. Assays were conducted at least in triplicate.

### Immunofluorescence (IF)

For transfection, SCC-25 cells at a density of 3 × 10^5^ were seeded in 30 mm cell culture dishes on coverslips with reduced-serum Opti-MEM (Gibco). The cells were transfected with 50 nM siRNA-HIF-1α or siRNA-Par3 at 37 °C under normoxic or hypoxic conditions as mentioned above for 24 h. After treatment, the cells were fixed in 4% PFA at room temperature for 20 min, and permeabilized in PBS with 0.5% Triton X-100. Nonspecific reactivity was blocked by preincubation in 10% normal goat serum in PBS. The cells were incubated with primary antibodies for Par3 (R&D Systems, 1:100 dilution) and TJP1 (Novus Biologicals, 1:100 dilution) at 4 °C overnight. After washing with PBS, the cover slips were incubated with corresponding secondary antibodies labeled with Alexa Fluor-488 or Alexa Fluor-568 (Invitrogen) for 2 h. Then, the coverslips were washed with PBS and mounted with Fluoroshield Mounting Medium With DAPI (Abcam). Images were acquired using a confocal microscope (Leica) with a 63× oil objective lens. Z-stacks were analyzed in three confocal slices and merged using ImageJ (Z-stack/maximum projection) to obtain the entire depth distribution.

### Immunohistochemistry (IHC)

Tissues were fixed in 4% buffered formalin (Sigma) for 24 h, embedded in paraffin, and sectioned. After deparaffinization and dehydration with xylene, the sections were rehydrated in serial dilutions of ethanol. Then, they were incubated in 3% H_2_O_2_ at room temperature for 20 min, washed with PBS, incubated in an antigen retrieval solution (sodium sulfate buffer, pH 6.0), and cooled to room temperature. To block nonspecific activity, the sections were incubated with 3% BSA, followed by overnight incubation with antibodies for HIF-1α (GeneTex, Inc., 1:1,000 dilution) and Par3 (R&D Systems, 1:1,000 dilutions). After washing with PBS, the sections were incubated with biotinylated goat anti-rabbit or anti-mouse IgG (Cell Signaling Technology, 1:5,000 dilution) for 1 h, washed with PBS, and incubated with the substrate diaminobenzidine (Abcam). Staining was observed using inverted microscopy. The sections were then counterstained with hematoxylin (BBC Biochemical, Mt. Vernon, WA, USA) and washed with water. After dehydration with serial dilutions of ethanol and xylene, the samples were sealed with a mixture of Canada balsam (Sigma-Aldrich). Staining intensity was scored from zero to two: grade 0, none; grade 1 < 25; and grade 2, ≥ 25 positively stained cells (Additional file 1: Fig. S1). Final scores were independently determined by two pathologists who were blinded to the clinical patient information. For statistical analysis, patients were divided into low- and high-expression groups based on median expression levels.

### In vivo analysis of tumor growth and tumor metastasis

All animal experiments were performed at Gangeung-Wonju National University according to the relevant guidelines. The experiments were approved by the Ethics Committee on Animal Experiments of Gangneung-Wonju National University (permit number GWNU-2020-36-1). For xenograft analysis, OSCC cells (2 × 10^6^ cells in 100 µL of Dulbecco’s PBS) were injected subcutaneously into 5-week-old male BALB/c nude mice. Tumor growth was monitored and measured weekly using tumor calipers. Tumor volumes were calculated as 0.52 × width^2^ × length. When the tumors reached a volume of approximately 1.5 mm^3^, the mice were killed and the tumors were collected and quickly frozen in liquid nitrogen for mRNA expression analysis.

To study lung metastasis in vivo, the cytoplasmic membranes of OSCC cells (1 × 10^6^ cells in 100 µL of Dulbecco’s PBS) were labeled with 10 µM non-cytotoxic Vybrant™ DiD Cell-Labeling Solution (Thermo Fisher Scientific) at 37 °C in the presence of 5% CO_2_ for 20 min right before intravenous OSCC cell injection into the tail veins of 8-week-old male BALB/c nude mice. After five weeks, metastasis in the lungs was detected using the FUSION Solo S imaging system with a SPECTRA capsule (Vilber). Excitation and emission filters were used to detect DiD-positive cells at 640 and 670 nm, respectively. All results were acquired at the same size using the auto exposure option of the instrument without any user input. Bioluminescence images were acquired and analyzed using automatic normalization in the Fusion acquisition software (Vilber). The signal was expressed as the number of photons emitted per second. For quantification of lung metastasis, images were captured and the diameters of tumor foci were measured using the cellSens standard software (Olympus). Following imaging, part of the lungs was quickly frozen in liquid nitrogen for mRNA expression analysis and part was formalin-fixed for hematoxylin and eosin (H&E) staining.

### Statistical analysis

All assays were repeated at least three times, and the data are presented as mean ± standard error of the mean. The significance of differences in the test variables between the control and experimental groups were analyzed using one-way analysis of variance or Student’s *t*-test. Correlations between clinicopathological parameters and scores of tumor IHC staining were analyzed using Levene’s test and the Mann–Whitney test. Statistical significance was set at *P* < 0.05. For the mouse experiments, six mice were assigned to each group. Statistical analysis was performed using SPSS (version 20.0; SPSS, Inc., Chicago, IL, USA) and GraphPad Prism 9 (GraphPad Software, San Diego, CA, USA).

## Results

### HIF-1α is overexpressed in OSCC tissues and is positively correlated with metastatic potential

**Fig. 1:**
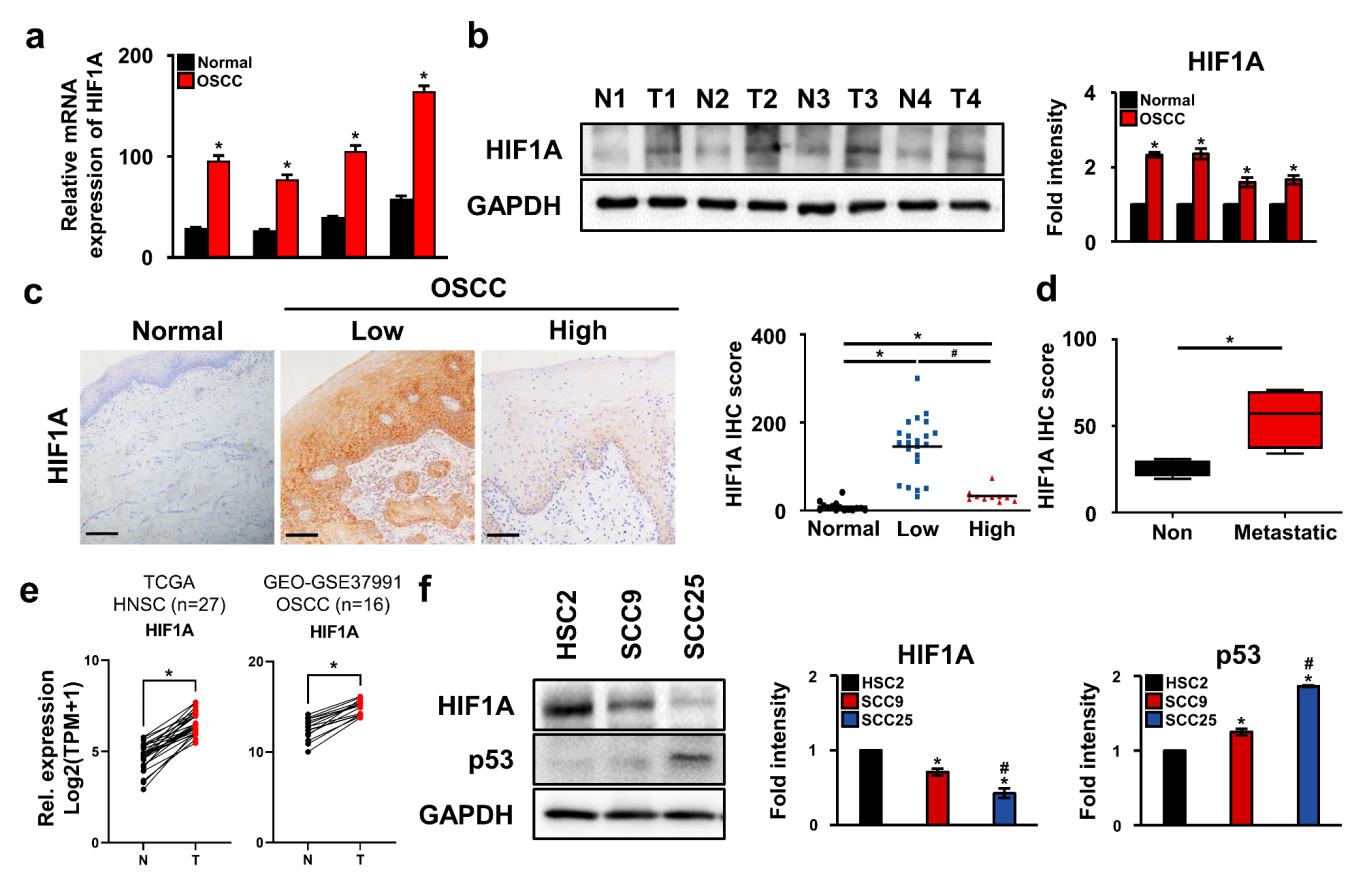
HIF-1α expression in OSCC tissues and cell lines. **a** mRNA and **b** protein levels of HIF-1α in matched OSCC and adjacent non-tumorous tissues as determined by qRT-PCR and western blotting, respectively. *P < 0.05 vs. normal tissues. The experiments were performed in triplicates. **c** Representative images of IHC staining of HIF-1α expression in OSCC tissues and normal tissues. Scale bars: 100 μm. HIF-1α expression scores are shown in the dot plot. Different grades of OSCC tissues and matched normal tissues were compared using a paired Student’s *t*-test. n = 29. *P < 0.05 vs. normal tissues. #*P* < 0.05 vs. low-grade OSCC tissues. **d** HIF-1α expression scores are shown as bar graph. Metastatic and non-metastatic OSCC tissues were compared using a paired Student’s t-test. n = 12. *P < 0.05 vs. non-metastatic tissues. The experiments were performed in triplicates. **e** HIF-1α mRNA levels in tumor and adjacent non-tumorous tissues of HNSC (left, TCGA) and OSCC (right, GEO-GSE37991). *P < 0.05 vs. normal tissues. **f** Western blot of protein levels of HIF-1α in OSCC cell lines. *P < 0.05 vs. HSC-2 cells. ^#^*P* < 0.05 vs. SCC-9 cells. The experiments were performed in triplicates. N: non-tumorous tissues; T: tumor tissues; Normal: non-tumorous tissues; Low: low-grade OSCC tissues; High: high-grade OSCC tissues; Non: non-metastatic OSCC tissues; Metastatic: metastatic OSCC tissues

To investigate the role of hypoxia in OSCC progression, we first analyzed HIF-1α expression in paired OSCC tumor and adjacent normal tissues. HIF-1α mRNA and protein levels were significantly upregulated in OSCC tissues compared to those in normal tissues (Fig. 1a, b). IHC results corroborated that HIF-1α expression was increased in OSCC tumor tissues compared to that in normal tissues, and it was strongly detected in the cytoplasm of epithelial cells. Quantitative analysis further demonstrated that HIF-1α expression was significantly higher in tumor tissues than in normal tissues; however, HIF-1α was rarely expressed in high-grade OSCC tissues compared to that in low-grade OSCC tissues (Fig. 1c). Moreover, HIF-1α mRNA levels were significantly increased in OSCC tumor tissues from patients with metastasis, indicating a positive correlation between HIF-1α expression and OSCC metastasis (Fig. 1d). HIF-1α was found to be significantly overexpressed in HNSC and OSCC in a public database (Fig. 1e). Next, we examined HIF-1α expression in three oral cancer cell lines: HSC-2 (metastatic site), SCC-9 (tongue), and SCC-25 (tongue). HIF-1α expression levels were higher in HSC-2 cells than in SCC-9 and SCC-25 cells, and the lowest in SCC-25 cells. In contrast, the expression level of p53 (a tumor suppressor marker) was the lowest in HSC-2 cells and the highest in SCC-25 cells (Fig. 1f). Taken together, these results suggested a potential role for HIF-1α not only in primary tumors, but also in the metastatic progression of OSCC.

### Hypoxia regulates the expression and localization of Par3, which affects the metastatic properties of OSCC cell lines

**Fig. 2:**
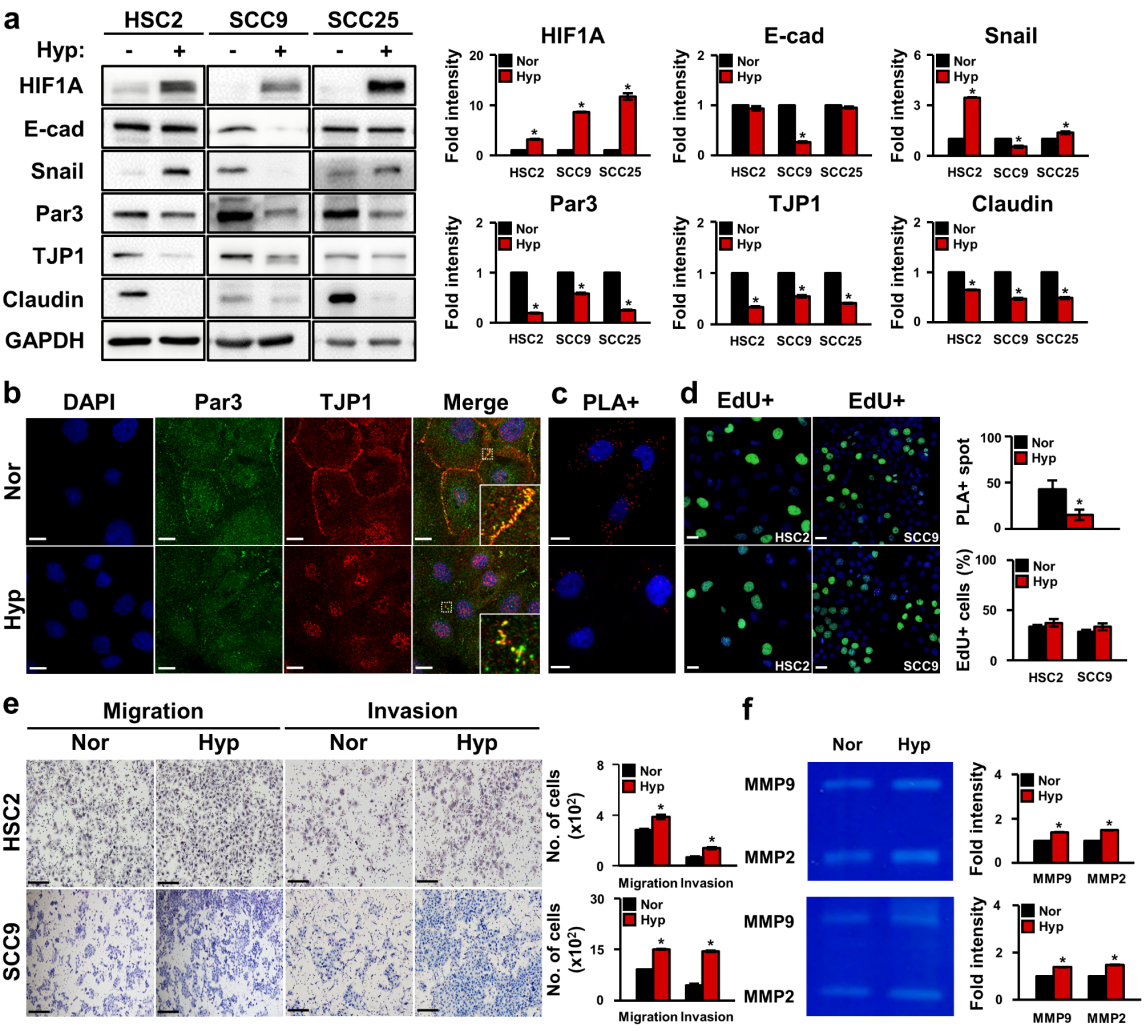
Effect of hypoxia on AJC marker expression in OSCC cell lines. **a** Western blot of protein levels (left) and quantification (right) of junction proteins in OSCC cell lines under normoxic and hypoxic conditions. The experiments were performed at least thrice. **b** Localization of Par3 (Green) and TJP1 (Red) in SCC-25 cell line under normoxic and hypoxic conditions as analyzed by IF staining. White boxes are magnified images of the dashed line boxes. Scale bars: 20 μm. The experiments were performed in triplicates. **c** Interaction between Par3 (green) and TJP1 (red) in SCC-25 cell line under normoxic and hypoxic conditions as analyzed by in situ PLA. Red spots represent PLA positive signals. Scale bars: 20 μm. The experiments were performed in triplicates. **d** Cell proliferation ability of OSCC cells under normoxic and hypoxic conditions as analyzed by the EdU assay. Green colored cells are EdU-positive. Scale bars: 20 μm. Nuclei were stained with DAPI (blue). The experiments were performed at least thrice. **e** Representative images (left) and numbers (right) of migrated and invaded OSCC cells under normoxic and hypoxic conditions. Scale bars: 100 μm. The experiments were performed at least thrice. **f** Activities (left) and fold intensity (right) of MMP-9 and MMP-2 in OSCC cells under normoxic and hypoxic conditions as analyzed by gelatin zymography. *P < 0.05 vs. normoxic conditions. The experiments were performed in at least thrice. Nor: normoxic conditions; Hyp: hypoxic conditions; PLA+: PLA positive signals in OSCC cells; EdU+: EdU-positive cells

The microenvironment of solid tumors is often hypoxic and plays an important role in alterations in cell–cell adhesion, cell polarity, and the barrier between epithelial cells [34, 35]. To investigate the effects of hypoxia on protein expression in OSCC cells, we incubated the three OSCC cell lines under normoxic or hypoxic conditions for one day and then quantified the protein expression of HIF-1α and junction proteins. HIF-1α expression was significantly increased under hypoxic conditions compared to that under normoxic conditions in all three cell lines. The expression of E-cadherin, a marker of adherens junctions, was unaffected in HSC-2 and SCC-25 cells, but was significantly decreased in SCC-9 cells under hypoxic conditions compared to that under normoxic conditions. The expression of Snail, a marker of the mesenchymal phenotype, was significantly increased in HSC-2 and SCC-25 cells, but was significantly decreased in SCC-9 cells under hypoxic conditions compared to that under normoxic conditions in all three cell lines. In all three OSCC cell lines, the expression of Par3 and TJ proteins (TJ protein 1 (TJP1) and claudin) was significantly decreased in hypoxic conditions compared to that under normoxic conditions (Fig. 2a).

In general, Par3 is found at the apical border of epithelial cells, such as TJs, and plays a role in the positioning of the apical barrier [36]. To investigate whether hypoxia regulates the positioning of the apical barrier in OSCC cells, we stained SCC-25 cells for Par3 and TJP1. These cells are a useful model for studying epithelial cell junctions because they form hyper-keratinized cells [37]. IF staining revealed that Par3 co-localized with TJP1 in the apical region of cell–cell contacts in cells cultured under normoxic conditions. However, localization of TJP1 and Par3 was disrupted in cells under hypoxic conditions (Fig. 2b). We further investigated the direct interaction between TJP1 and Par3 in the cell–cell contact region using an in situ PLA. Ligation signals were detected in cell–cell contact regions in cells cultured under normoxic conditions, but were diminished under hypoxic conditions (Fig. 2c). These results indicated that hypoxia may regulate the expression and localization of junction proteins, including TJP1 and Par3, as well as the positioning of the apical barrier in OSCC cells.

EdU assay results revealed that the proliferation ability of OSCC cells was not significantly affected by culture in hypoxic conditions (Fig. 2d). However, the migration and invasion abilities of HSC-2 and SCC-9 cells were significantly increased under hypoxic conditions compared to those under normoxic conditions (Fig. 2e). MMPs are the main enzymes involved in the degradation of collagen and other proteins in the extracellular matrix (ECM) during tumor growth and metastasis, and they determine the levels of migration and invasion in cancer cells [38]. We previously demonstrated that MMP-2 and MMP-9 activities are important for the invasion ability of OSCC cells [33]. Therefore, we performed gelatin zymography to analyze MMP-2 and MMP-9 activities in HSC-2 and SCC-9 cells under normoxic and hypoxic conditions. MMP-2 and MMP-9 activities were found to be significantly higher in both cell lines under hypoxic conditions compared to those under normoxic conditions (Fig. 2f). Collectively, these findings suggested that hypoxia promotes OSCC cell migration and invasion via MMP-2 and MMP-9 activities.

### Knockdown of HIF-1α affects the protein expression and localization of Par3 and TJs in OSCC cell lines

**Fig. 3:**
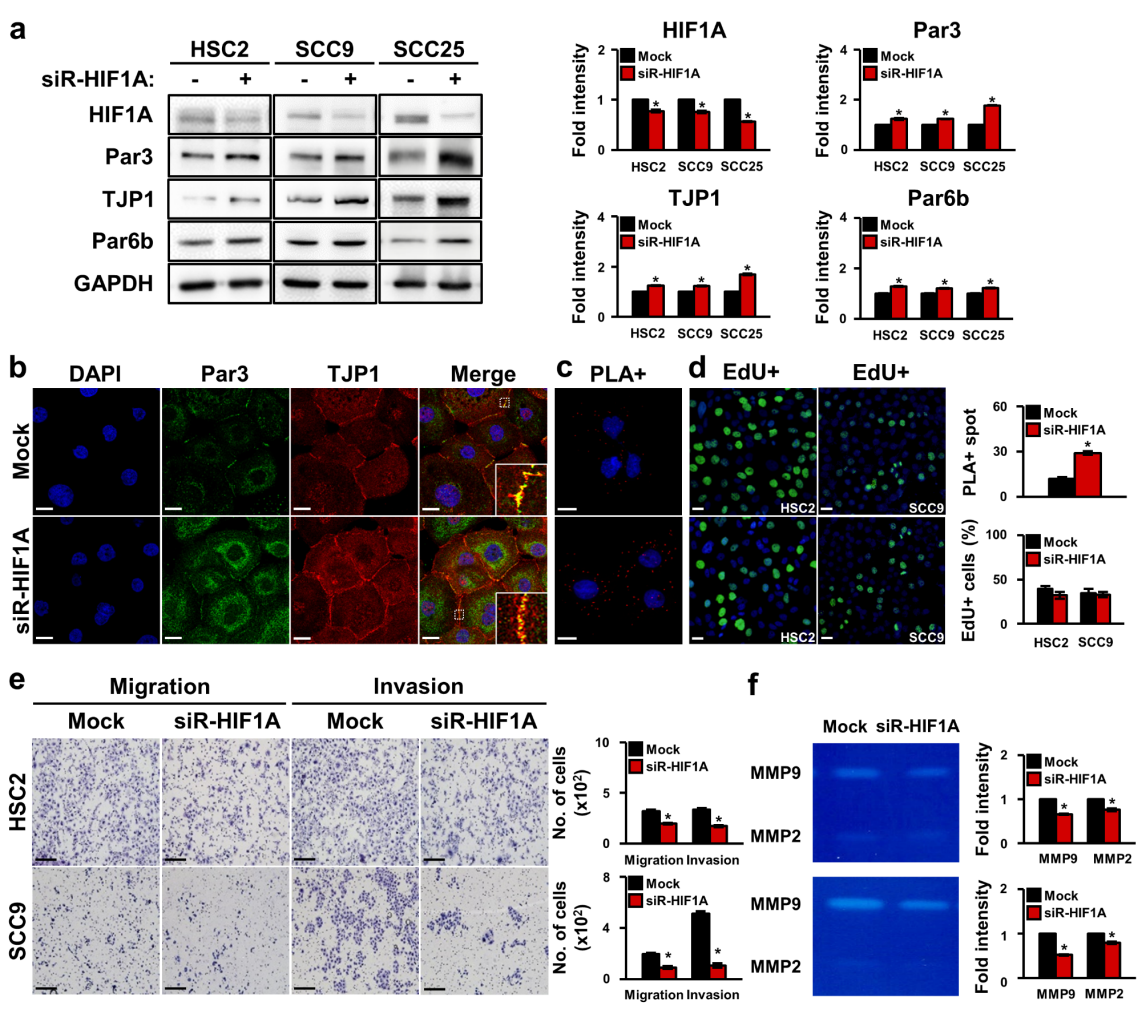
Effect of HIF-1α on AJC marker expression in OSCC cell lines. **a** Western blot of protein levels (left) and quantification (right) of AJC markers in OSCC cell lines treated with siRNA-HIF-1α or control siRNA. The experiments were performed in triplicates. **b** Localization of Par3 (Green) and TJP1 (Red) in SCC-25 cell line treated with siRNA-HIF-1α or control siRNA as analyzed by IF staining. White boxes are magnified images of the dashed line boxes. Scale bars: 20 μm. The experiments were performed in triplicates. **c** Interaction between Par3 and TJP1 in SCC-25 cell line treated with siRNA-HIF-1α or control siRNA as analyzed by in situ PLA. Red spots represent PLA positive signals. The experiments were performed in triplicates. **d** Cell proliferation ability of OSCC cells treated with siRNA-HIF-1α or control siRNA as analyzed by the EdU assay. Green colored cells are EdU-positive cells. Scale bars: 20 μm. The nuclei were stained with DAPI (blue). The experiments were performed at least thrice. **e** Representative images (left) and numbers (right) of migrated and invaded OSCC cells treated with siRNA-HIF-1α or control siRNA. Scale bars: 100 μm. The experiments were performed at least thrice. **f** Activities (left) and fold intensities (right) of MMP-9 and MMP-2 in OSCC cells treated with siRNA-HIF-1α or control siRNA as analyzed by gelatin zymography. *P < 0.05 vs. control siRNA. The experiments were performed in triplicates. Cont: control siRNA; siR-HIF1A: siRNA- HIF-1α; Hyp: hypoxia; PLA+: PLA positive signals in OSCC cells; EdU+: EdU-positive cells

The above results demonstrated that the hypoxic microenvironment is crucial factor for the regulation of expression and localization of TJ proteins in the apical cell–cell contact region. Hypoxia-induced HIF-1α regulates the expression of various target genes. Therefore, we reasoned that HIF-1α may play a crucial role in the gene expression of Par3 and TJs. To determine whether HIF-1α inhibition directly regulates the expression of Par3 and TJs in OSCC, we knocked down HIF-1α in the OSCC cell lines using siRNAs. We found that Par3, TJP1 and Par6b protein levels were significantly increased in all three OSCC cells upon HIF-1α silencing (Fig. 3a). IF staining results revealed that Par3 and TJP1 were more strongly localized in apical cell–cell contact regions in the HIF-1α knockdown group than in the control group (Fig. 3b). Furthermore, the PLA signal was significantly increased in the HIF-1α knockdown group compared to that in the control group (Fig. 3c). These results suggested that the interaction between Par3 and TJP1 depends on HIF-1α.

Next, we investigated the proliferation ability of the OSCC cell lines in response to HIF-1α expression. EdU assay results revealed that the proliferation ability of OSCC cells was not significantly affected by HIF-1α knockdown (Fig. 3d). Transwell assays showed that the migration and invasion abilities were significantly decreased in HIF-1α knockdown cells compared to those in control (Fig. 3e). In gelatin zymography, HIF-1α knockdown significantly suppressed MMP-2 and MMP-9 activities, indicating the importance of HIF-1α-mediated MMP activity in OSCC cells (Fig. 3f). These results suggested that HIF-1α directly regulates the migration and invasion abilities of OSCC cells via MMP-2 and MMP-9 activities.

### Par3 knockdown affects TJ expression in OSCC cells

**Fig. 4:**
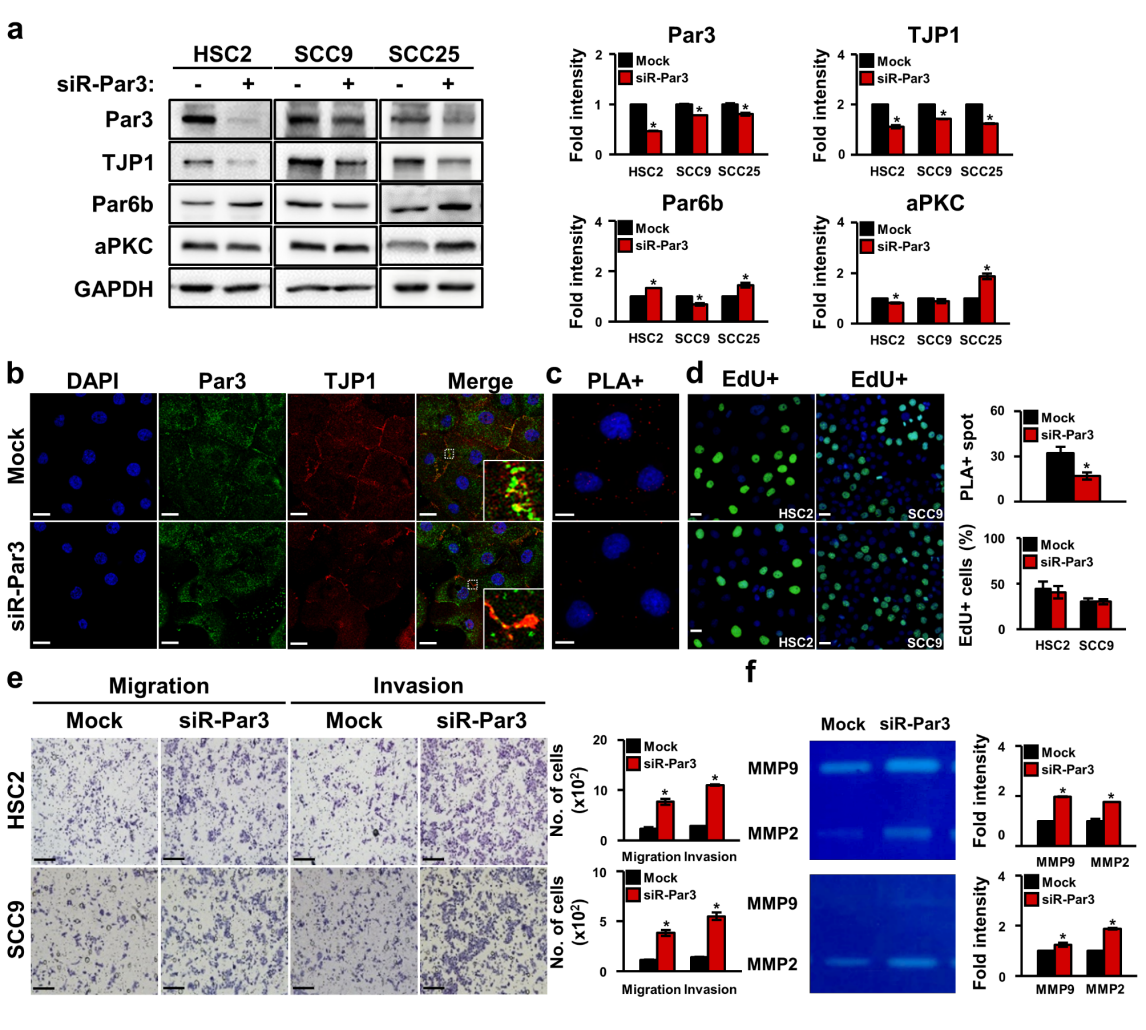
Effect of Par3 on AJC marker expression in OSCC cells. **a** Western blot of protein levels (left) and quantification (right) of AJC markers in OSCC cell lines treated with siRNA-Par3 or control siRNA. The experiments were performed in triplicates. **b** Localization of Par3 (Green) and TJP1 (Red) in SCC-25 cell line treated with siRNA-Par3 or control siRNA as analyzed by IF staining. Scale bars: 20 μm. The experiments were performed in triplicates. **c** Interaction between TJP1 and Par3 in SCC-25 cell line treated with siRNA-Par3 or control siRNA. Red spots represent PLA positive signals. Scale bars: 20 μm. The experiments were performed in triplicates. **d** Proliferation ability of OSCC cells treated with siRNA-Par3 or control siRNA as analyzed by the EdU assay. Green colored cells are EdU-positive cells. The nuclei were stained with DAPI (blue). Scale bars: 20 μm. The experiments were performed at least thrice. **e** Representative images (left) and numbers (right) of migrated and invaded OSCC cells treated with siRNA-Par3 or control siRNA. The experiments were performed at least thrice. Scale bars: 100 μm. **f** Activities (left) and fold intensities (right) of MMP-9 and MMP-2 in OSCC cells treated with siRNA-Par3 or control siRNA as analyzed by gelatin zymography. *P < 0.05 vs. control siRNA. The experiments were performed in triplicates. Cont: control siRNA; siR-Par3: siRNA-Par3; Hyp: hypoxia; PLA+: PLA positive signals in OSCC cells; EdU+: EdU-positive cells

We knocked down Par3 in the OSCC cell lines to provide direct experimental evidence that hypoxia mediates Par3 regulation of the expression and apical position of TJs in OSCC. TJP1 expression was universally reduced when Par3 expression was reduced in three OSCC cell lines although there were individually differences in expression of Par6b and aPKC among three OSCC cell lines (Fig. 4a). Microscopic studies showed that the localization of Par3 and TJP1 was significantly disrupted in the Par3 knockdown group compared to that in the control group, despite the presence of a weak TJP1 signal (Fig. 4b, magnified images). In PLA, the ligation signal was significantly decreased upon reduction of Par3 (Fig. 4c). These results suggested that TJ disruption in OSCC is directly regulated by Par3 expression. EdU assay results revealed no significant difference in the proliferation ability of OSCC cells after Par3 knockdown (Fig. 4d). Par3 has been reported to be involved in tumor metastasis [39]. We hypothesized that hypoxia may affect OSCC metastatic properties via Par3. To test this hypothesis, we investigated the migration and invasion abilities of OSCC cells transfected with siRNA-Par3 in comparison to cells transfected with control siRNA. The results showed that after Par3 knockdown, OSCC migration and invasion were significantly increased (Fig. 4e). As expected, MMP-2 and MMP-9 activities were significantly increased in Par3 knockdown cells compared to those in control cells (Fig. 4f). Taken together, these results revealed that hypoxia promotes the migration and invasion abilities of OSCC cells via MMP activity by inactivating Par3 expression.

### Functional role of HIF-1α in OSCC in vivo

**Fig. 5:**
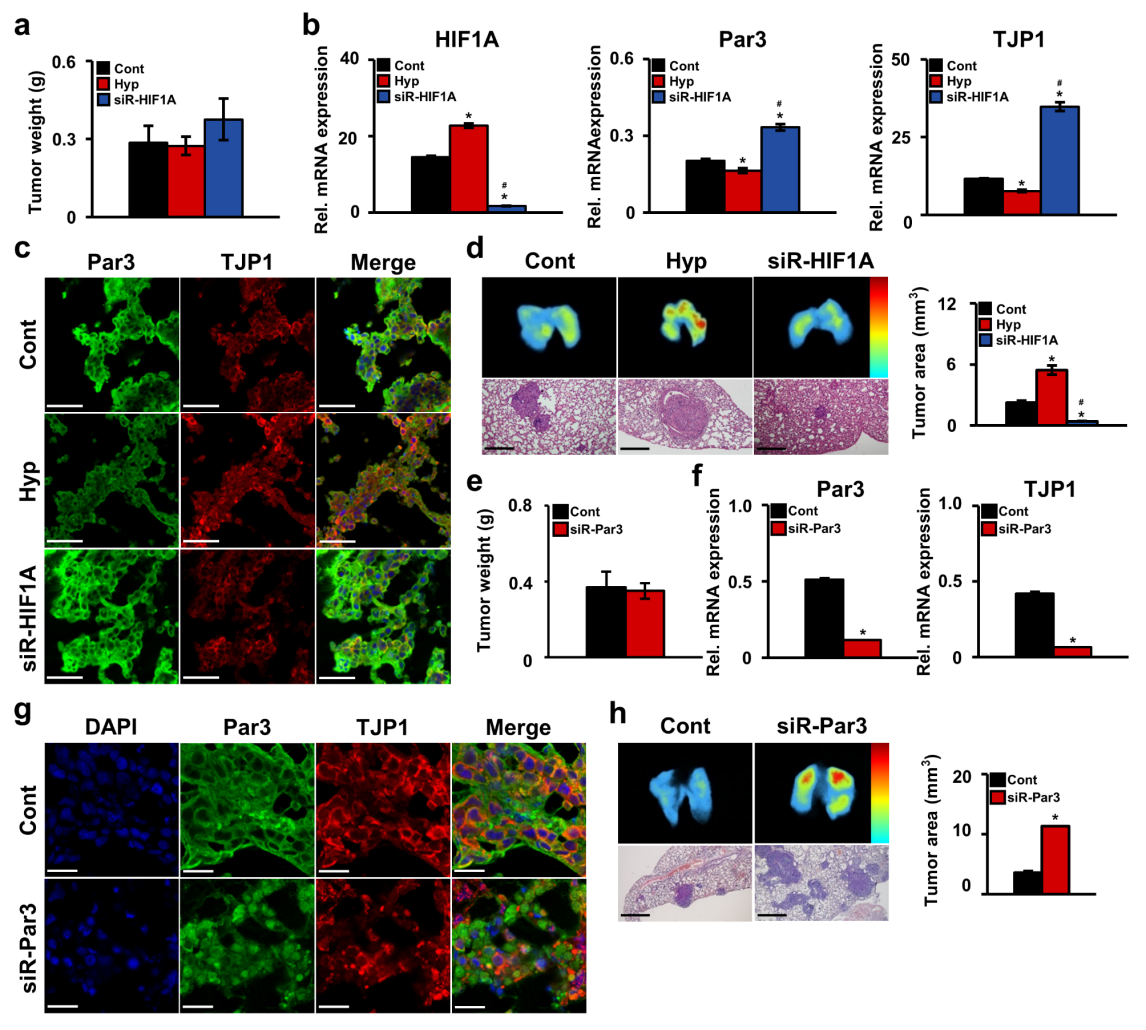
Effects of HIF-1α and Par3 on OSCC tumor metastasis in vivo. HSC-2 cells treated with normoxia and control siRNA (Cont), hypoxia (Hyp), or siRNA-HIF-1α or siRNA-Par3 were injected into BALB/c nude mice. **a** Xenograft tumor weights. **b** mRNA levels of HIF-1α, Par3, and TJP1 as analyzed by qRT-PCR. The experiments were performed at least thrice. **c** Localization of Par3 and TJP1 in mice xenografts as analyzed by IF staining. The nuclei were stained with DAPI (blue). Scale bars: 20 μm. The experiments were performed at least thrice. **d** Lung metastasis as analyzed by quantifying the DiD signal (upper panels) and tumor area (lower panels). The color gradient indicates the intensity of bioluminescence (red = intense, blue = not intense). The diameters of tumor foci in the lungs were determined after H&E staining. *P < 0.05 vs. normoxic conditions. #*P* < 0.05 vs. hypoxic condition. The experiments were performed in triplicates. **e** Xenograft tumor weights. **f** mRNA levels of Par3 and TJP1 as analyzed by qRT-PCR. The experiments were performed at least thrice. **g** Localization of Par3 and TJP1 in mouse xenografts as analyzed by IF staining. The nuclei were stained with DAPI (blue). Scale bars: 20 μm. The experiments were performed at least thrice. **h** Lung metastasis as analyzed by quantifying the DiD signal (upper panels) and tumor area (lower panels). The color gradient indicates the intensity of bioluminescence (red = intense, blue = not intense). The diameters of tumor foci in the lungs were determined after H&E staining. The experiments were performed in triplicates. *P < 0.05 vs. control

To investigate the role of HIF-1α in tumor growth in vivo, HSC-2 cells treated with normoxia and control siRNA (control group), hypoxia (hypoxia group), or siRNA-HIF-1α (siR-HIF-1α group) were subcutaneously injected into BALB/c nude mice. The size and weight of the xenograft tumors did not differ among the groups (Fig. 5a and Additional file 1: Fig. S2a, b). However, the mRNA levels of Par3 and TJP1 were significantly changed depend on HIF-1α expression, indicating that Par3 and TJP1 are negatively regulated by HIF-1α (Fig. 5b). Furthermore, IF staining revealed that TJ and Par3 localization was significantly disrupted in the hypoxia group, and that it was strongly detected in the cell–cell contact region in the siR-HIF-1α group compared to that in the control group (Fig. 5c). To investigate the functional effect of HIF-1α on tumor metastasis in vivo, DiD-labeled HSC-2 cells treated with normoxia and siRNA (control group), hypoxia (hypoxia group), or siRNA-HIF-1α (siR-HIF-1α group) were intravenously injected into nude mice to establish lung metastasis. The bioluminescent signal was significantly higher in the hypoxia group than in the control group, whereas it was significantly lower in the siR-HIF-1α group than in the control group (Fig. 5d, upper panels). In addition, H&E staining revealed that the lung metastasis areas in the hypoxia group were significantly larger than those in the control group, but significantly smaller in the siR-HIF-1α group than in the control group (Fig. 5d, lower panels). These results suggested that HIF-1α expression is positively correlated with OSCC cell metastasis in vivo.

### Functional role of Par3 in the metastatic potential of OSCC in vivo

According to publicly available data, Par3 expression is lower in HNSC and OSCC than in normal tissues (Additional file 1: Fig. 3S3a, b). Further, it is lower in OSCC metastatic tissues than in adjacent normal tissues (Additional file 1: Fig. S3c, d). To investigate the functional effect of Par3 on tumor growth in vivo, HSC-2 cells treated with normoxia and control siRNA (control group) or siRNA-Par3 (siR-Par3 group) were subcutaneously injected into nude mice. The tumor size and weight of xenograft tumors did not differ between the siR-Par3 and control groups (Fig. 5e and Additional file 1: Fig. S4a, b). TJP1 mRNA expression was significantly decreased in the siR-Par3 group, indicating that TJP1 is positively regulated by Par3 (Fig. 5f). Furthermore, the localization of TJP1 and Par3 was significantly disrupted in the siR-Par3 group compared to that in the control group (Fig. 5g). To investigate the effect of Par3 on tumor metastasis in vivo, DiD-labeled HSC-2 cells were intravenously injected to establish lung metastasis. The bioluminescent signal was significantly higher in the siR-Par3 group than in the control group (Fig. 5h, upper panels). In addition, the lung metastatic areas in the siR-Par3 group were significantly larger than those in the control group (Fig. 5h lower panels). These results showed that Par3 expression was negatively correlated with the metastatic properties of OSCC cells in vivo.

## Discussion

Metastasis and recurrence are the most common prognostic factors of OSCC [40]. Therefore, it is critical to investigate the molecular mechanisms of metastasis and tumorigenesis in order to identify novel targets for prevention and treatment. In this study, we investigated the role of hypoxia in OSCC progression and metastasis. We demonstrated that HIF-1α is expressed at higher levels in OSCC tissues than in adjacent non-tumor tissues. However, HIF-1α expression was significantly higher in low-grade OSCC tissue than in high-grade OSCC tissue. In general, HIF-1α overexpression is associated with a poor prognosis and tumor progression [41, 42]. However, the clinical correlation between HIF-1α and tumor prognosis and progression in OSCC remains controversial. Some studies have shown that HIF-1α expression is upregulated in superficial OSCC and is associated with the early stage of OSCC transformation and development, suggesting that HIF-1α is a cancer risk marker [43, 44]. Furthermore, Ribeiro et al. demonstrated that high HIF-1α expression in OSCC tumor margins is critical in the transition from primary lesions to invasive cancer [45]. We previously found that HIF-1α expression is higher in metastatic OSCC tissues than in non-metastatic OSCC tissues [46]. These findings suggested that HIF-1α may facilitate regional metastasis from precursor lesions.

To provide direct experimental evidence that HIF-1α regulates OSCC metastasis and growth, we first measured endogenous expression levels of HIF-1α in three OSCC cell lines, HSC-2, SCC-9, and SCC-25, in vitro. HIF-1α expression was found to be significantly higher in HSC-2 cells than in SCC-9 and SCC-25 cells. Morphologically, there are some differences among the three cell lines, although all cells have the typical shape of squamous cell carcinoma. For example, HSC-2 cells are round to oval tumor cells without cell–cell contact, whereas SCC-9 and SCC-25 cells are polygonal with cell–cell junctions (Additional file 1: Fig. S5a). The HIF-1α-dependent differences were also reflected in the invasion ability of the cells; the invasion ability of HSC-2 cells was significantly higher than that of the other cell lines, whereas that of SCC-25 cells rarely showed invasion (Additional file 1: Fig. S5b). Therefore, SCC-25 cells were used to detect the localization of the TJ proteins in the present study. Hypoxia is a physiological cue that determines cell proliferation and metastatic properties. Generally, hypoxia acts as a stressor. Therefore, tumor cells migrate away from the primary tumor region rather than proliferate, which further increases the oxygen demand [47]. Hence, we investigated the molecular mechanism of hypoxia-regulated metastatic potential in OSCC.

Cell–cell junctions of epithelial cells are made up of the apical junctional complex (AJC), including TJs, which maintain cell and tissue homeostasis through mechanical stability. However, abnormal AJC structure is frequently observed in tumors of epithelial origin, and TJ disruption can lead to invasion into adjacent tissues and metastasis [48, 49]. Although loss of TJs has been observed to be involved in metastasis, whether and how it plays a role in OSCC metastasis is unclear. Hypoxia has also been shown to alter AJC expression and localization, resulting in EMT and cancer metastasis [18, 50–52]. However, it is unclear whether there is a link between hypoxia, TJs, and metastasis in OSCC. Despite the fact that EMT was observed in some experiments in the present study, we found that hypoxia alone was not sufficient to induce EMT in all OSCC cell lines. Previous studies have shown that alteration of HIF-1α alone is also not sufficient to induce EMT [53, 54], suggesting that EMT is not uniform and that other mechanisms are involved in hypoxia-induced AJC disruption. It has been reported that Par3 can regulate the TJs [55, 56], but research on the underlying mechanism is limited. Furthermore, the relationship between the expression of Par3 and TJs in OSCC is not fully understood. In this study, we first demonstrated that hypoxia reduces Par3 expression and localization, which results in TJ disruption, leads to OSCC invasion. We next questioned what the underlying mechanisms are those by which the mislocalization of Par3 triggers OSCC invasion under hypoxia. Par3 normally regulates TJ formation by interacting with other components of the polarity complex (e.g., Par6b and PKCζ) at the apical junction region. Recently, Zhou et al. showed that in non-small-cell lung cancer, hypoxia induces the degradation of Par3/PKCζ/Par6, which contributes to tumor invasion via EMT [26]. Suzuki et al. reported that the interaction between Par3 and Par6 is indirect and is mediated by PKCζ [57]. In contrast to findings in other studies, we found that hypoxia does not affect Par6 expression in OSCC, and the interactions of Par3 with Par6 and PKC are not fully understood. Nevertheless, our results suggest that Par3 localizes to TJs and that their interaction is disrupted when OSCC cells are exposed to hypoxia.

MMPs are a family of zinc- and calcium-dependent enzymes initially identified as ECM components. Based on their ability to degrade ECM, MMPs are classified into collagenases, gelatinases, matrilysins, and stromelysins [58]. However, the role of MMPs is not limited to the degradation of the ECM and that of other substrates. Vermeer et al. recently showed that MMP-9 cleaves cell–cell junctions, and apical MMP-9 treatment resulted in epithelial barrier disruption via loss of TJ integrity, claudin-1, and occludin, leading to alteration of the cell architecture [59]. Various MMPs are strongly expressed in patients with OSCC, with MMP-2, -8, -9, and − 13 being particularly associated with OSCC progression and depth of invasion into adjacent tissue [60]. MMP-2 and − 9 have been shown to regulate the invasion ability of OSCC cells [33]. Together, these results suggest that MMP-2 and − 9 may modulate TJ integrity to induce OSCC cell invasion. We found that hypoxia enhanced the invasion ability of OSCC cells by promoting MMP-2 and MMP-9 activities, whereas HIF-1α inhibition reduced the invasion ability of OSCC cells by inhibiting these enzyme activities. Some studies have suggested that the Par3–Stat3 and Par3–DDR1 signaling pathways regulate cell–cell junction integrity and the invasion ability of cancer cells via MMP-9 and membrane type 1-MMP, respectively [30, 61].

This study has some limitations. Firstly, qRT-PCR, western blot and IHC analysis was performed in a small number of OSCC patients. The large-scale cohort is needed to validate the relationship between HIF-1α and OSCC metastasis. Secondly, although the function of HIF-1α and Par3 activities was confirmed in OSCC cell lines in vitro, animal studies should be conducted to elucidate this mechanism as well as how they directly or indirectly regulates MMP-2 and MMP-9 activities.

## Conclusions

In summary, our results revealed that HIF-1α expression was upregulated in patients with OSCC and that it was closely associated with the invasion ability of OSCC cells (HSC-2 and SCC-9 cell lines) under hypoxia. Additionally, the interaction between Par3 and TJ proteins was disrupted by hypoxia in OSCC, accompanied by upregulation of MMP activity and metastatic properties of OSCC cells (SCC-25 cell line, Additional file 1: Fig. S6). Overall, our study highlights that the hypoxia-induced loss of expression of Par3 and TJ proteins may be a novel biomarker for OSCC metastasis. Furthermore, these findings may aid in elucidating the molecular mechanisms of OSCC metastasis and progression and in developing new diagnostic and therapeutic approaches for OSCC metastasis.

## Electronic supplementary material

Below is the link to the electronic supplementary material.

**Additional file 1: Table S1.** Clinicopathological features of 29 patients with OSCC, and association between Par3 expression these variables. **Additional file 1: Fig. S1.** The expression pattern of HIF-1α in OSCC. The IHC scores were classified as negative, low and high expression. **Additional file 1: Fig. S2.** The expression of HIF-1α has nothing to do with tumor growth in vivo. HSC-2 cells treated with or without hypoxic conditions and siRNA-HIF-1α were subcutaneously injected into the BALB/c nude mice. a The image and b growth curve of tumor volume was measured in mice xenografts. **Additional file 1: Fig. S3.** The expression of Par3 is negatively correlated with metastatic properties of OSCC. The mRNA expression of Par3 were significantly increased in patient cohort with a HNSC and b OSCC, compared with paired normal tissue. Results were retrieved from TCGA and GSE37991, respectively. Each lines represented paired adjacent normal tissues. * indicates significance differences between tumor and normal tissues (*P < 0.05*). c The mRNA and d protein expression of Par3 were decreased in metastatic patient cohort and tissues of OSCC, compared with non-metastatic OSCC tissues. Patient cohort was retrieved from GSE2880 (n = 14). The black box indicates cropped fields of original images. * indicates significance differences between metastatic and non-metastatic OSCC (*P < 0.05*). **Additional file 1: Fig. S4.** The expression of Par3 has nothing to do with tumor growth in vivo. HSC-2 cells treated with or without siRNA-Par3 were subcutaneously injected into the BALB/c nude mice. c The image and d growth curve of tumor volume was measured in mice xenografts. **Additional file 1: Fig. S5.** Morphologic changes are reflected in invasion ability of the OSCC cell lines. a The image represents the phase-contrast images of cell morphology of HSC-2, SCC-9 and SCC-25 cell lines. Scale bar, 100 μm. b the image represents the invaded number of HSC-2, SCC-9 and SCC-25 cells from upper chamber by matrigel invasion assay. **Additional file 1: Fig. S6**. A graphic model for the role of hypoxic conditions in OSCC. Hypoxic conditions are involved with disruption of tight junctions and promotes the metastatic properties of OSCC by Par3.

**Additional file 2: Figure S7**. The original figure of western blots of Fig. 1b. The red box indicated the representative bands of protein levels in Fig. 1b. Protein expression of HIF-1α in normal and OSCC patient tissues by western blotting. GAPDH was used as the internal control. **Additional file 2: Figure S8**. The original figure of western blots of Figure 1e. The red box indicated the representative bands of protein levels in Figure 1e. Protein expression of HIF-1α and p53 in OSCC cell lines by western blotting. GAPDH was used as the internal control. **Additional file 2: Figure S9**. The original figure of western blots of Figure 2a. The red box indicated the representative bands of protein levels in Fig. 2a. Protein expression of HIF-1α, E-cad, Snail, Par3, TJP1 and claudin in HSC-2 cell line under normoxia or hypoxic conditions by western blotting. GAPDH was used as the internal control. **Additional file 2: Figure S10**. The original figure of western blots of Figure 2a. The red box indicated the representative bands of protein levels in Fig. 2a. Protein expression of HIF-1α, E-cad, Snail, Par3, TJP1 and claudin in SCC-9 cell line under normoxia or hypoxic conditions by western blotting. GAPDH was used as the internal control. **Additional file 2: Figure S11**. The original figure of western blots of Figure 2a. The red box indicated the representative bands of protein levels in Fig. 2a. Protein expression of HIF-1α, E-cad, Snail, Par3, TJP1 and claudin in SCC-25 cell line under normoxia or hypoxic conditions by western blotting. GAPDH was used as the internal control. **Additional file 2: Figure S12**. The original figure of western blots of Figure 3a. The red box indicated the representative bands of protein levels in Fig. 3a. Protein expression of HIF-1α, Par3, TJP1 and Par6b in HSC-2 cell line treated with siRNA-HIF-1α or control siRNA by western blotting. GAPDH was used as the internal control. **Additional file 2: Figure S13**. The original figure of western blots of Figure 3a. The red box indicated the representative bands of protein levels in Fig. 3a. Protein expression of HIF-1α, Par3, TJP1 and Par6b in SCC-9 cell line treated with siRNA-HIF-1α or control siRNA by western blotting. GAPDH was used as the internal control. **Additional file 2: Figure S14**. The original figure of western blots of Figure 3a. The red box indicated the representative bands of protein levels in Fig. 3a. Protein expression of HIF-1α, Par3, TJP1 and Par6b in SCC-25 cell line treated with siRNA-HIF-1α or control siRNA by western blotting. GAPDH was used as the internal control. **Additional file 2: Figure S15**. The original figure of western blots of Figure 4a. The red box indicated the representative bands of protein levels in Fig. 4a. Protein expression of Par3, TJP1 Par6b and aPKC in HSC-2 cell line treated with siRNA-Par3 or control siRNA by western blotting. GAPDH was used as the internal control. **Additional file 2: Figure S16**. The original figure of western blots of Figure 4a. The red box indicated the representative bands of protein levels in Fig. 4a. Protein expression of Par3, TJP1 Par6b and aPKC in SCC-9 cell line treated with siRNA-Par3 or control siRNA by western blotting. GAPDH was used as the internal control. **Additional file 2: Figure S17**. The original figure of western blots of Figure 4a. The red box indicated the representative bands of protein levels in Fig. 4a. Protein expression of Par3, TJP1 Par6b and aPKC in SCC-25 cell line treated with siRNA-Par3 or control siRNA by western blotting. GAPDH was used as the internal control.

## Data Availability

The datasets used or analyzed during the present study are available from the corresponding author on reasonable request.

## List of abbreviations

AJC: Apical junctional complex
aPKC: Atypical protein kinase C
BSA: Bovine serum albumin
DAPI: 4′,6-diamidino-2-phenylindole
ECM: Extracellular matrix
EdU: 5-ethynyl-2′-deoxyuridine
EMT: Epithelial-to-mesenchymal transition
H&E: Hematoxylin and eosin
HIF-1α: Hypoxia-inducible factor 1-alpha
HNSC: Head and neck squamous cell carcinoma
IF: Immunofluorescence
IHC: Immunohistochemistry
MMP: Matrix metalloproteinase
OSCC: Oral squamous cell carcinoma
Par: Partitioning-defective
PBS: Phosphate-buffered saline
PBS-T: PBS with 0.01% Tween-20
PFA: Paraformaldehyde
PLA: Proximity ligation assay
qRT-PCR: Reverse transcription quantitative real-time polymerase chain reaction
SDS: Sodium dodecyl sulfate
siRNA: Small interfering RNA
TJs: Tight junctions
