# Supplementary material for: Hypoxia disrupt tight junctions and promote metastasis of oral squamous cell carcinoma via loss of par3
Source: Cancer Cell Int. 2023 Apr 24;23:79. doi: 10.1186/s12935-023-02924-8 (PMC10123966; doi:10.1186/s12935-023-02924-8)
Supplement: Supplementary file 1 — Additional file 1: Table S1. Clinicopathological features of 29 patients with OSCC, and association between Par3 expression these variables. Additional file 1: Fig. S1. The expression pattern of HIF-1α in OSCC. The IHC scores were classified as negative, low and high expression. Additional file 1: Fig. S2. The expression of HIF-1α has nothing to do with tumor growth in vivo. HSC-2 cells treated with or without hypoxic conditions and siRNA-HIF-1α were subcutaneously injected into the BALB/c nude mice. a The image and b growth curve of tumor volume was measured in mice xenografts. Additional file 1: Fig. S3. The expression of Par3 is negatively correlated with metastatic properties of OSCC. The mRNA expression of Par3 were significantly increased in patient cohort with a HNSC and b OSCC, compared with paired normal tissue. Results were retrieved from TCGA and GSE37991, respectively. Each lines represented paired adjacent normal tissues. * indicates significance differences between tumor and normal tissues (P < 0.05). c The mRNA and d protein expression of Par3 were decreased in metastatic patient cohort and tissues of OSCC, compared with non-metastatic OSCC tissues. Patient cohort was retrieved from GSE2880 (n = 14). The black box indicates cropped fields of original images. * indicates significance differences between metastatic and non-metastatic OSCC (P < 0.05). Additional file 1: Fig. S4. The expression of Par3 has nothing to do with tumor growth in vivo. HSC-2 cells treated with or without siRNA-Par3 were subcutaneously injected into the BALB/c nude mice. c The image and d growth curve of tumor volume was measured in mice xenografts. Additional file 1: Fig. S5. Morphologic changes are reflected in invasion ability of the OSCC cell lines. a The image represents the phase-contrast images of cell morphology of HSC-2, SCC-9 and SCC-25 cell lines. Scale bar, 100 μm. b the image represents the invaded number of HSC-2, SCC-9 and SCC-25 cells from upper chamber by matrig [file 12935_2023_2924_MOESM1_ESM.pptx]

## Slide 1
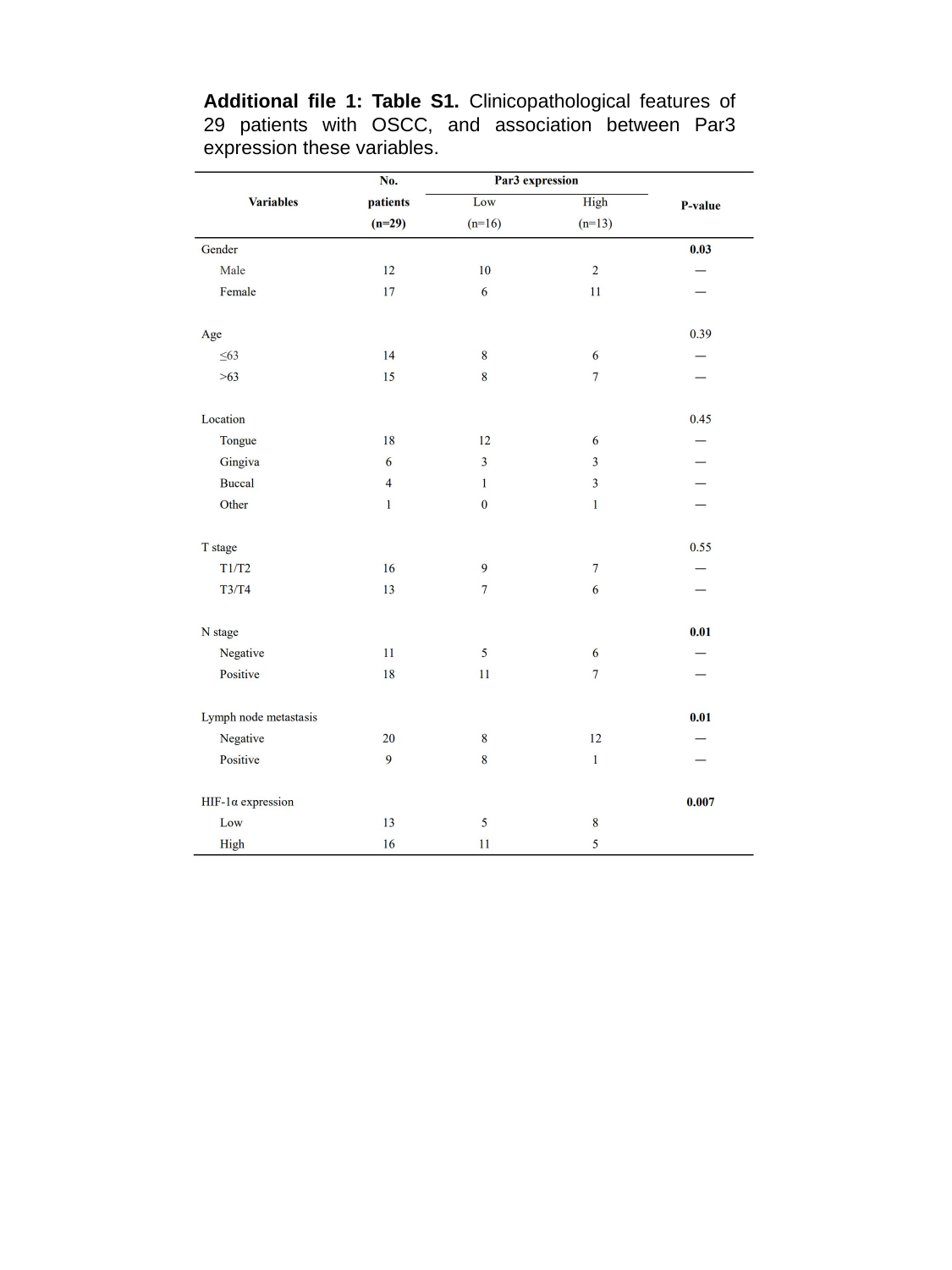

Additional file 1: Table S1. Clinicopathological features of 29 patients with OSCC, and association between Par3 expression these variables.

## Slide 2
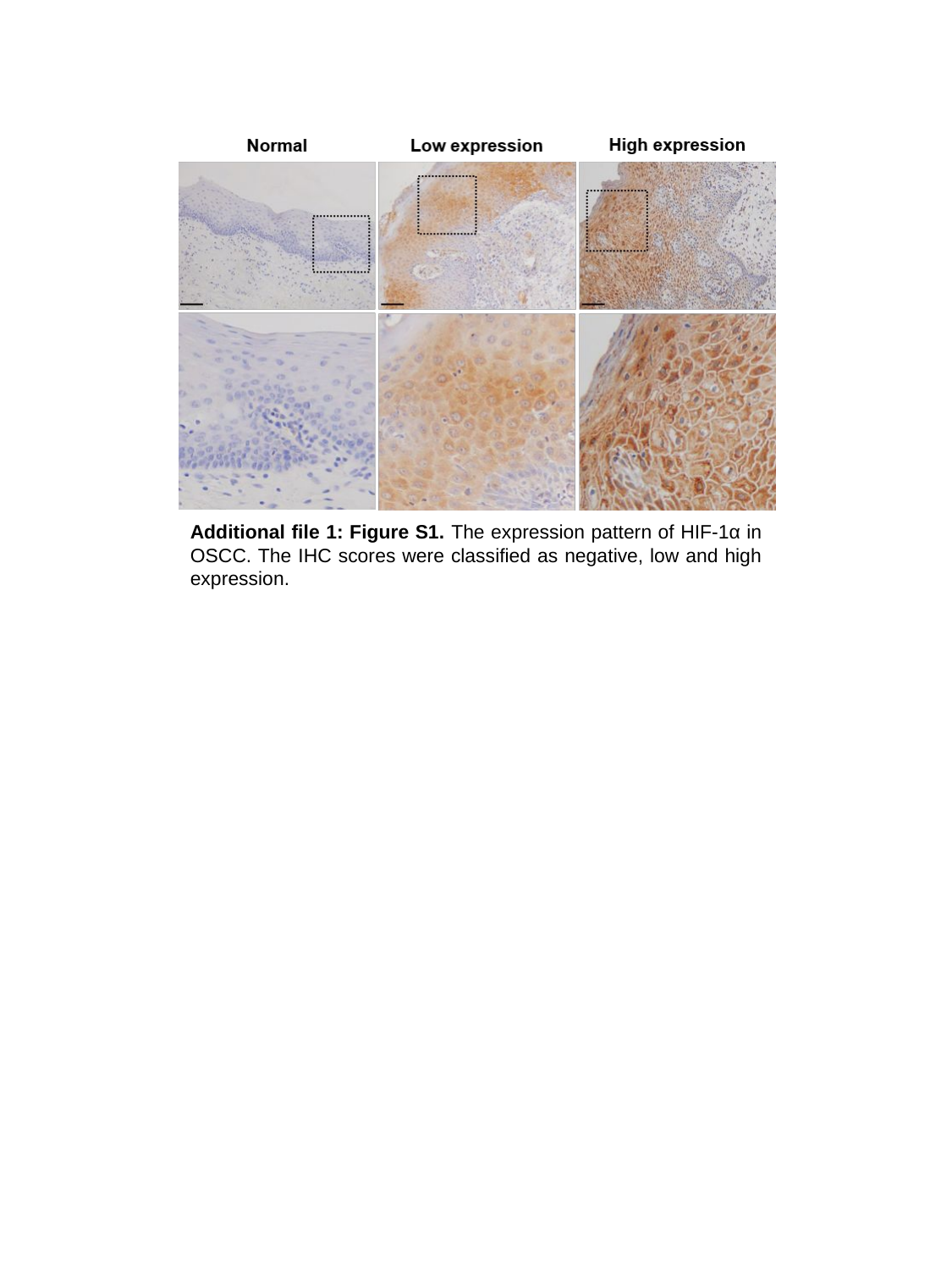

Additional file 1: Figure S1. The expression pattern of HIF-1α in OSCC. The IHC scores were classified as negative, low and high expression.

## Slide 3
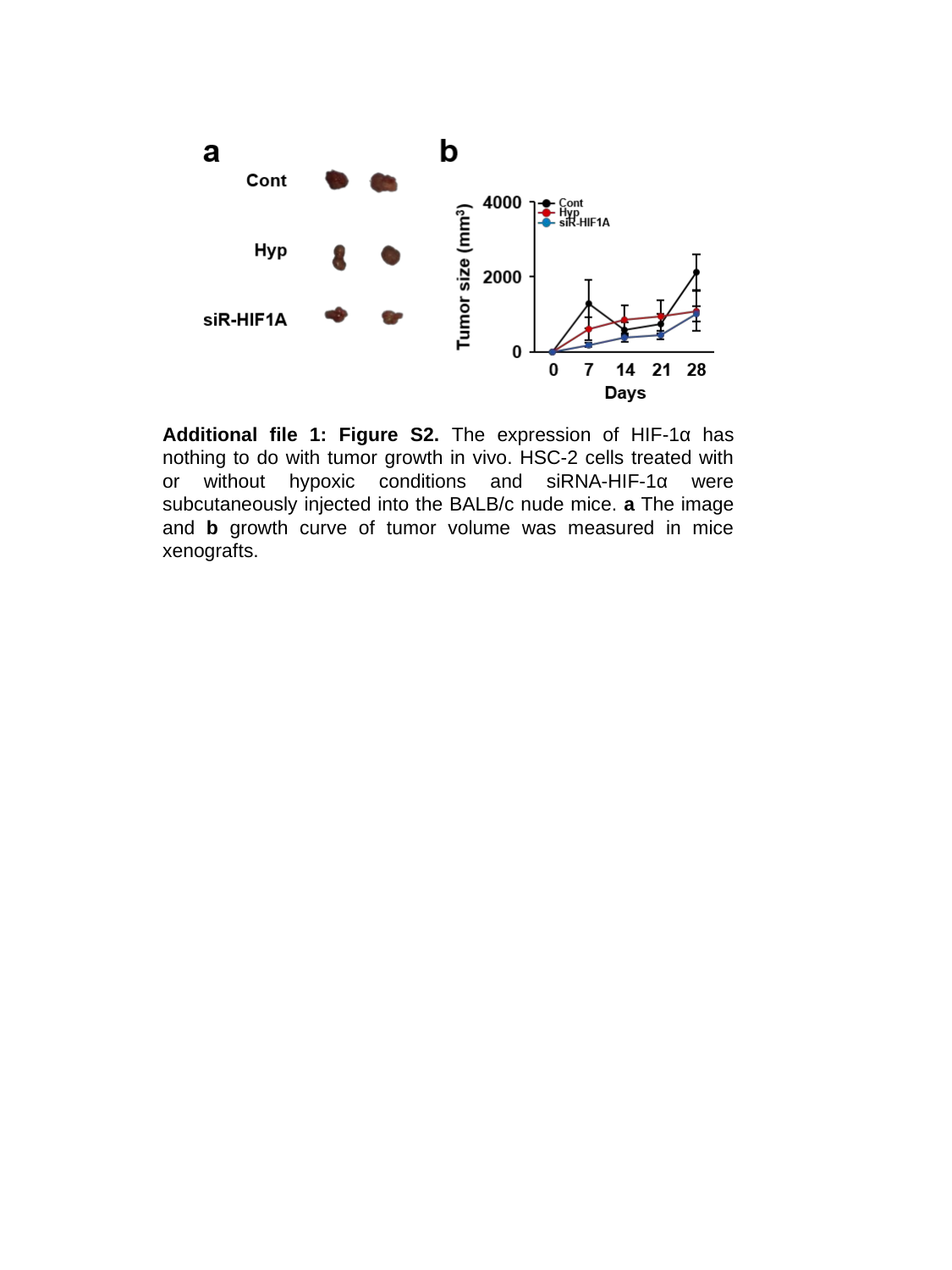

Additional file 1: Figure S2. The expression of HIF-1α has nothing to do with tumor growth in vivo. HSC-2 cells treated with or without hypoxic conditions and siRNA-HIF-1α were subcutaneously injected into the BALB/c nude mice. a The image and b growth curve of tumor volume was measured in mice xenografts.

## Slide 4
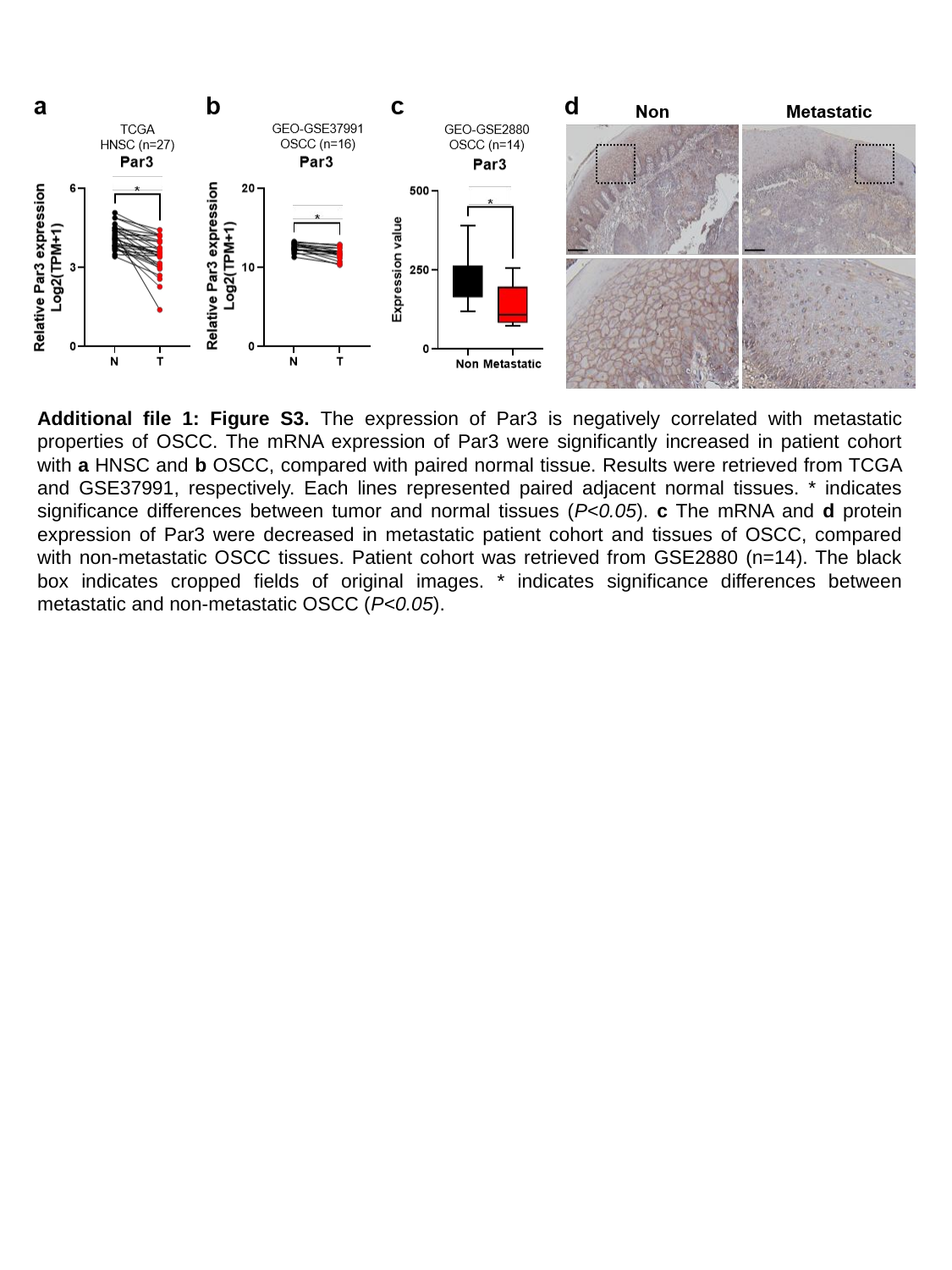

Additional file 1: Figure S3. The expression of Par3 is negatively correlated with metastatic properties of OSCC. The mRNA expression of Par3 were significantly increased in patient cohort with a HNSC and b OSCC, compared with paired normal tissue. Results were retrieved from TCGA and GSE37991, respectively. Each lines represented paired adjacent normal tissues. * indicates significance differences between tumor and normal tissues (P<0.05). c The mRNA and d protein expression of Par3 were decreased in metastatic patient cohort and tissues of OSCC, compared with non-metastatic OSCC tissues. Patient cohort was retrieved from GSE2880 (n=14). The black box indicates cropped fields of original images. * indicates significance differences between metastatic and non-metastatic OSCC (P<0.05).

## Slide 5
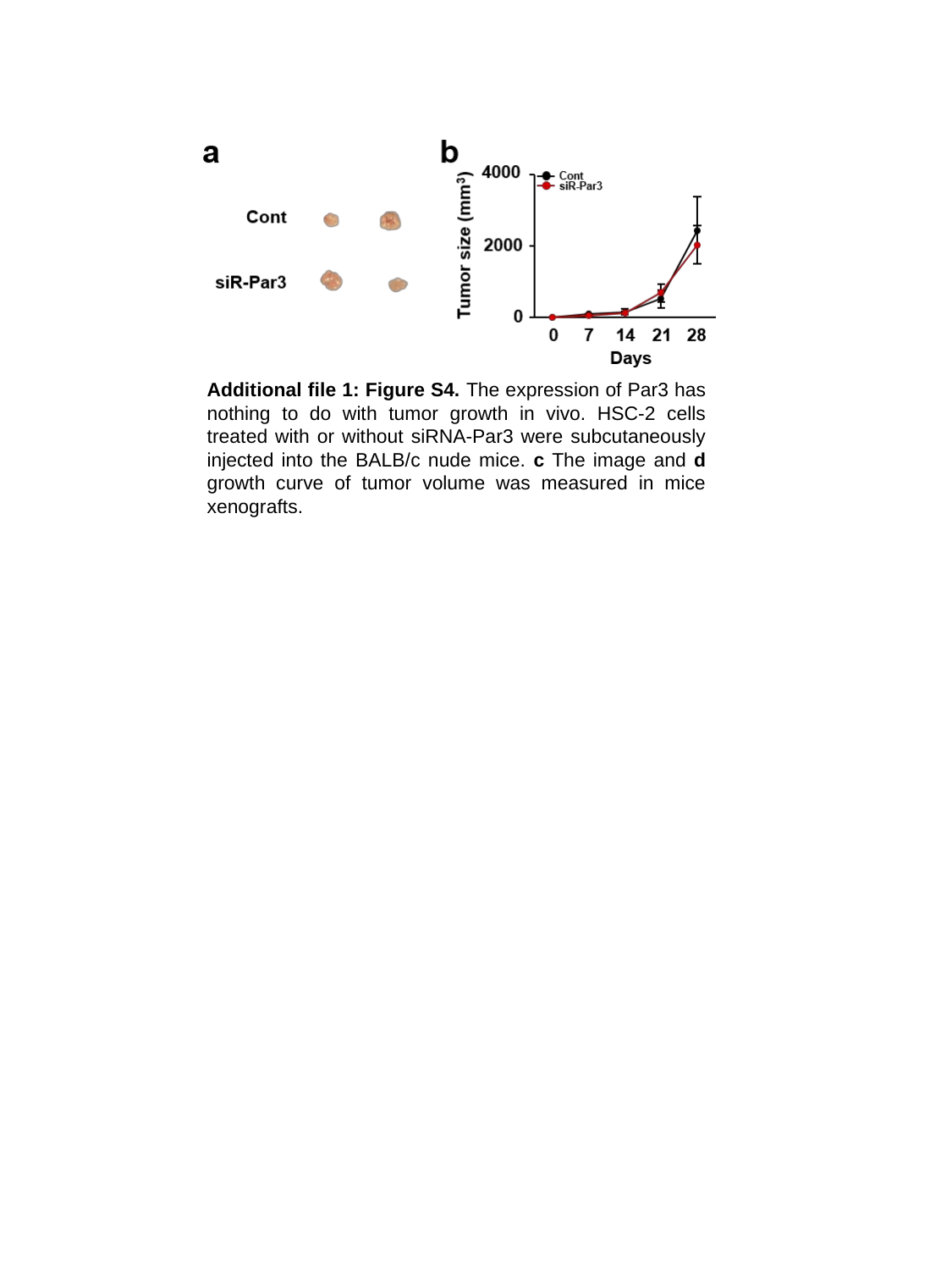

Additional file 1: Figure S4. The expression of Par3 has nothing to do with tumor growth in vivo. HSC-2 cells treated with or without siRNA-Par3 were subcutaneously injected into the BALB/c nude mice. c The image and d growth curve of tumor volume was measured in mice xenografts.

## Slide 6
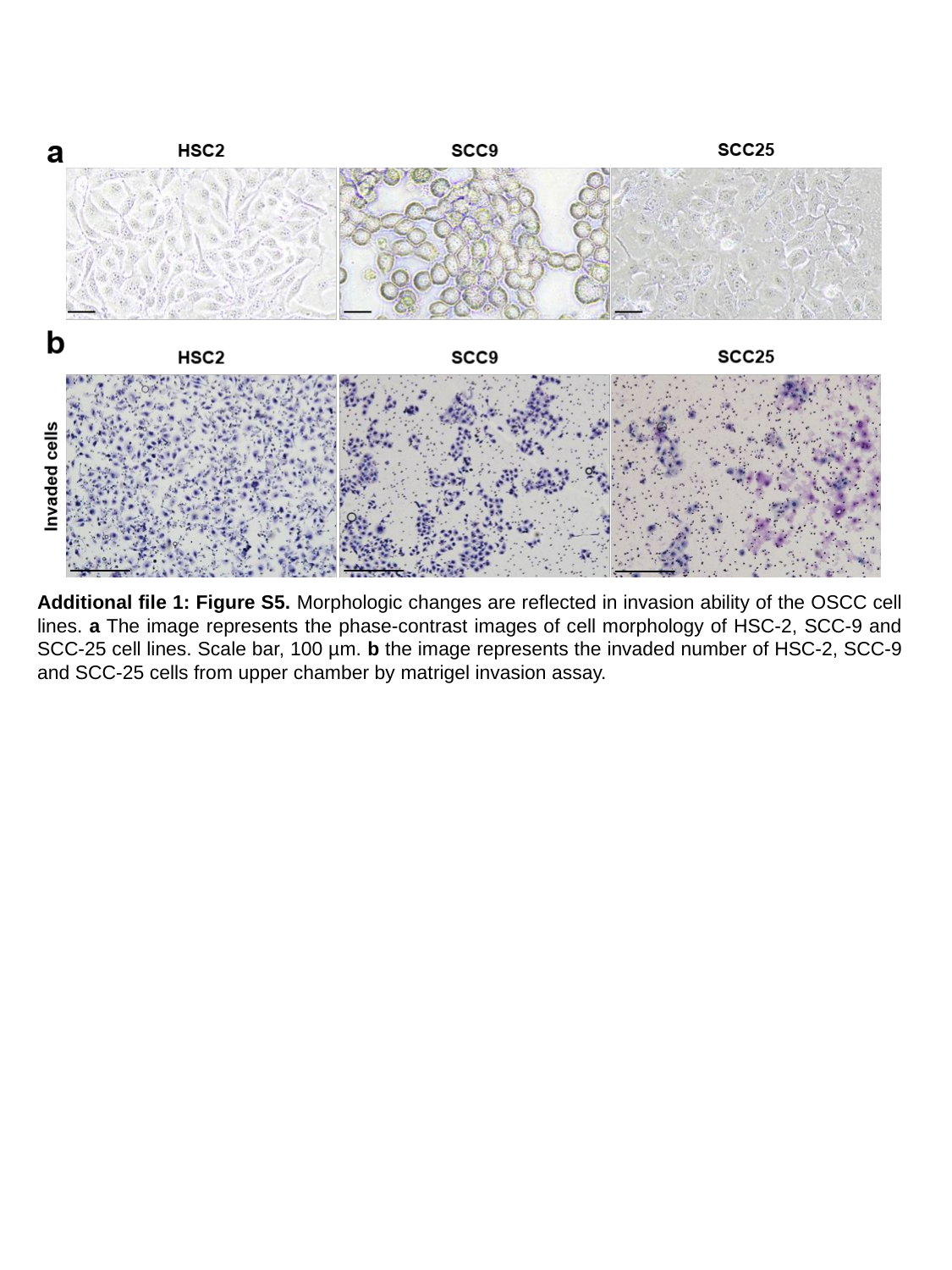

Additional file 1: Figure S5. Morphologic changes are reflected in invasion ability of the OSCC cell lines. a The image represents the phase-contrast images of cell morphology of HSC-2, SCC-9 and SCC-25 cell lines. Scale bar, 100 µm. b the image represents the invaded number of HSC-2, SCC-9 and SCC-25 cells from upper chamber by matrigel invasion assay.

## Slide 7
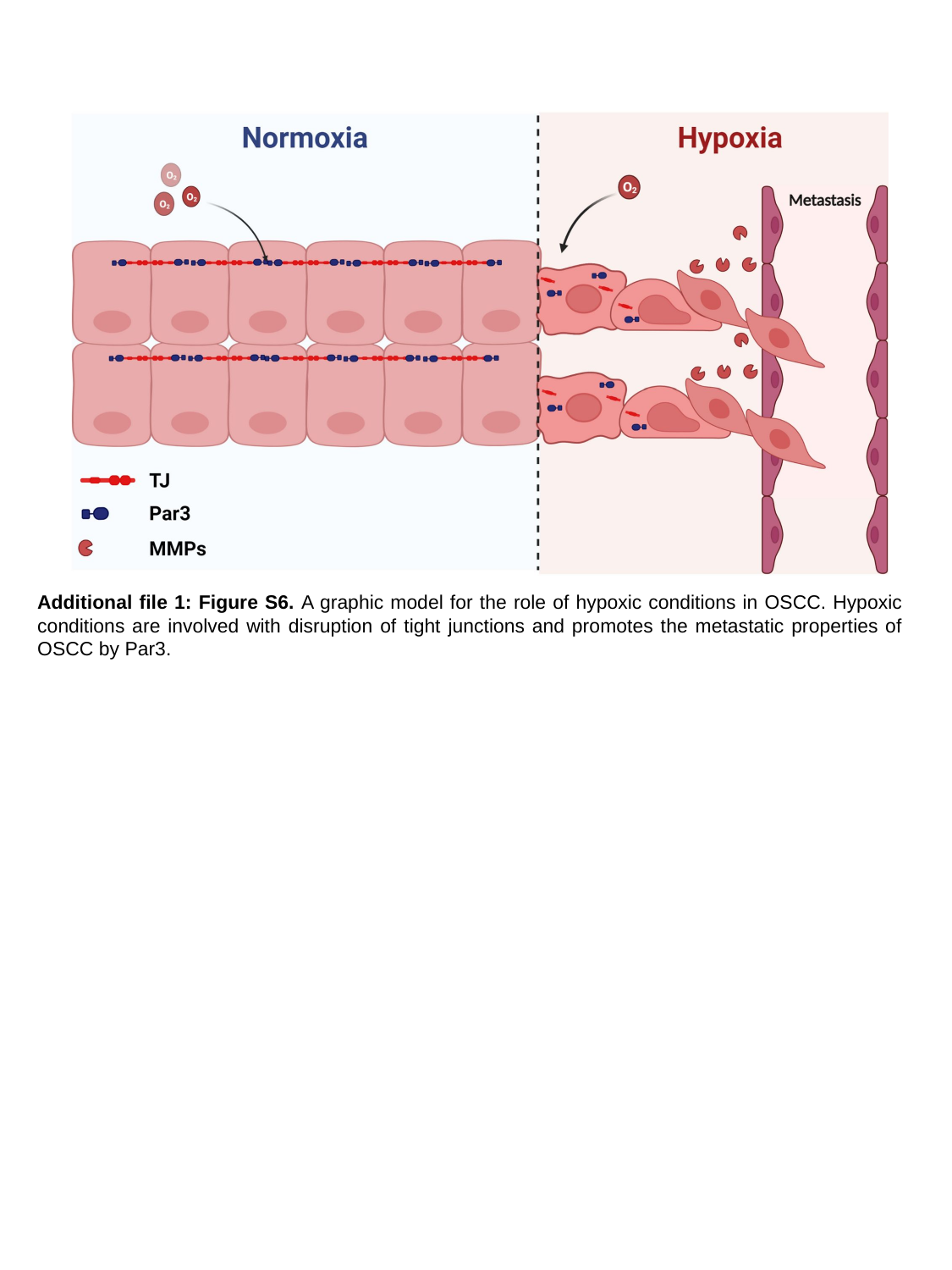

Additional file 1: Figure S6. A graphic model for the role of hypoxic conditions in OSCC. Hypoxic conditions are involved with disruption of tight junctions and promotes the metastatic properties of OSCC by Par3.
