# Supplementary material for: Hypoxia disrupt tight junctions and promote metastasis of oral squamous cell carcinoma via loss of par3
Source: Cancer Cell Int. 2023 Apr 24;23:79. doi: 10.1186/s12935-023-02924-8 (PMC10123966; doi:10.1186/s12935-023-02924-8)
Supplement: Supplementary file 2 — Additional file 2: Figure S7. The original figure of western blots of Fig. 1b. The red box indicated the representative bands of protein levels in Fig. 1b. Protein expression of HIF-1α in normal and OSCC patient tissues by western blotting. GAPDH was used as the internal control. Additional file 2: Figure S8. The original figure of western blots of Figure 1e. The red box indicated the representative bands of protein levels in Figure 1e. Protein expression of HIF-1α and p53 in OSCC cell lines by western blotting. GAPDH was used as the internal control. Additional file 2: Figure S9. The original figure of western blots of Figure 2a. The red box indicated the representative bands of protein levels in Fig. 2a. Protein expression of HIF-1α, E-cad, Snail, Par3, TJP1 and claudin in HSC-2 cell line under normoxia or hypoxic conditions by western blotting. GAPDH was used as the internal control. Additional file 2: Figure S10. The original figure of western blots of Figure 2a. The red box indicated the representative bands of protein levels in Fig. 2a. Protein expression of HIF-1α, E-cad, Snail, Par3, TJP1 and claudin in SCC-9 cell line under normoxia or hypoxic conditions by western blotting. GAPDH was used as the internal control. Additional file 2: Figure S11. The original figure of western blots of Figure 2a. The red box indicated the representative bands of protein levels in Fig. 2a. Protein expression of HIF-1α, E-cad, Snail, Par3, TJP1 and claudin in SCC-25 cell line under normoxia or hypoxic conditions by western blotting. GAPDH was used as the internal control. Additional file 2: Figure S12. The original figure of western blots of Figure 3a. The red box indicated the representative bands of protein levels in Fig. 3a. Protein expression of HIF-1α, Par3, TJP1 and Par6b in HSC-2 cell line treated with siRNA-HIF-1α or control siRNA by western blotting. GAPDH was used as the internal control. Additional file 2: Figure S13. The original figure of western blots of Figure 3a [file 12935_2023_2924_MOESM2_ESM.pptx]

## Slide 1
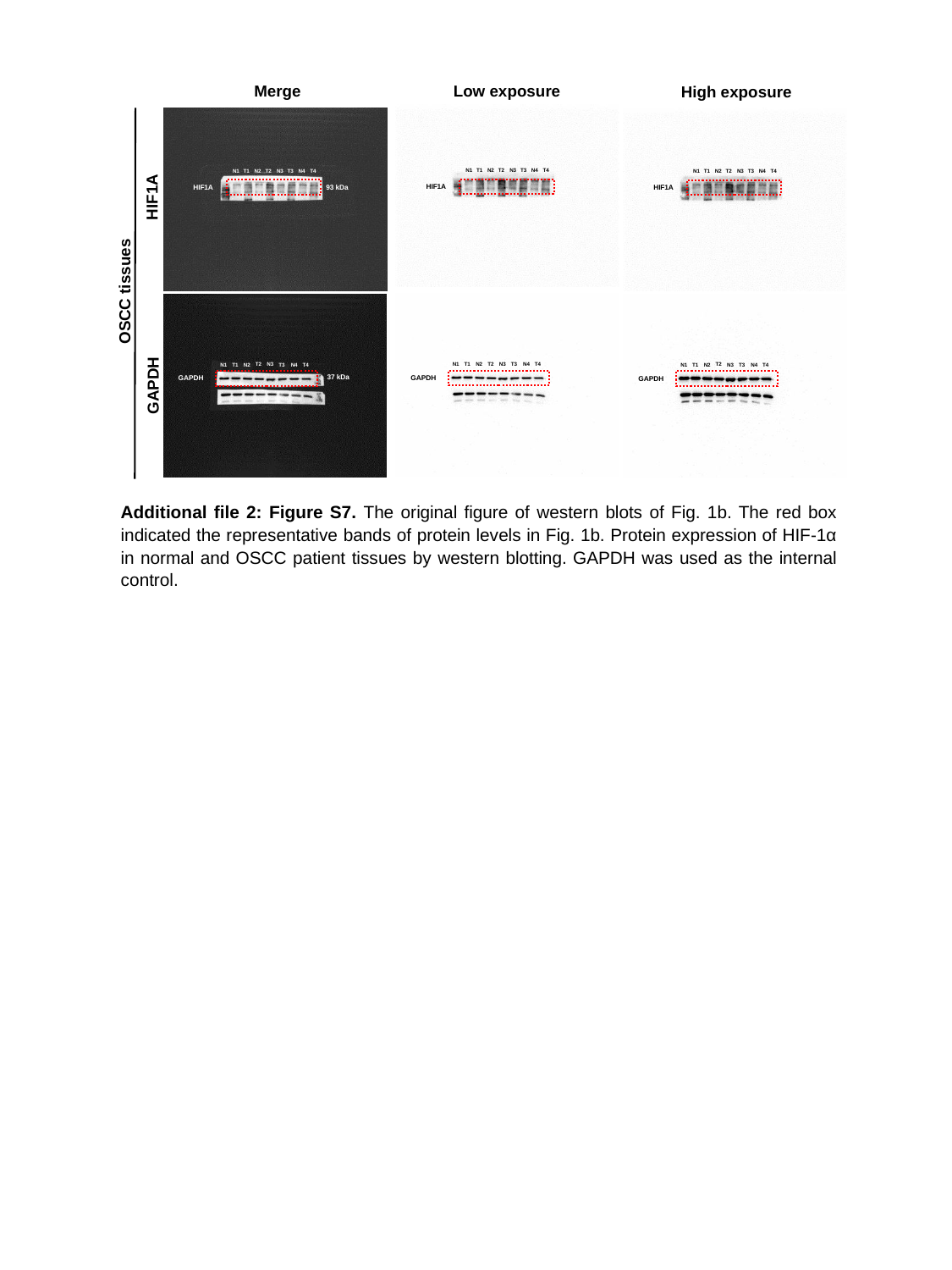

Merge
Low exposure
High exposure
T2
N3
T4
T1
N1
N2
N4
T3
T2
N3
T4
T1
N1
N2
N4
T3
T2
N3
T4
T1
N1
N2
N4
T3
HIF1A
93 kDa
HIF1A
HIF1A
HIF1A
OSCC tissues
T2
N3
T4
T1
N1
N2
N4
T3
T2
N3
T2
N3
T4
T1
T4
N1
N2
N4
T3
T1
N1
N2
N4
T3
37 kDa
GAPDH
GAPDH
GAPDH
GAPDH
Additional file 2: Figure S7. The original figure of western blots of Fig. 1b. The red box indicated the representative bands of protein levels in Fig. 1b. Protein expression of HIF-1α in normal and OSCC patient tissues by western blotting. GAPDH was used as the internal control.

## Slide 2
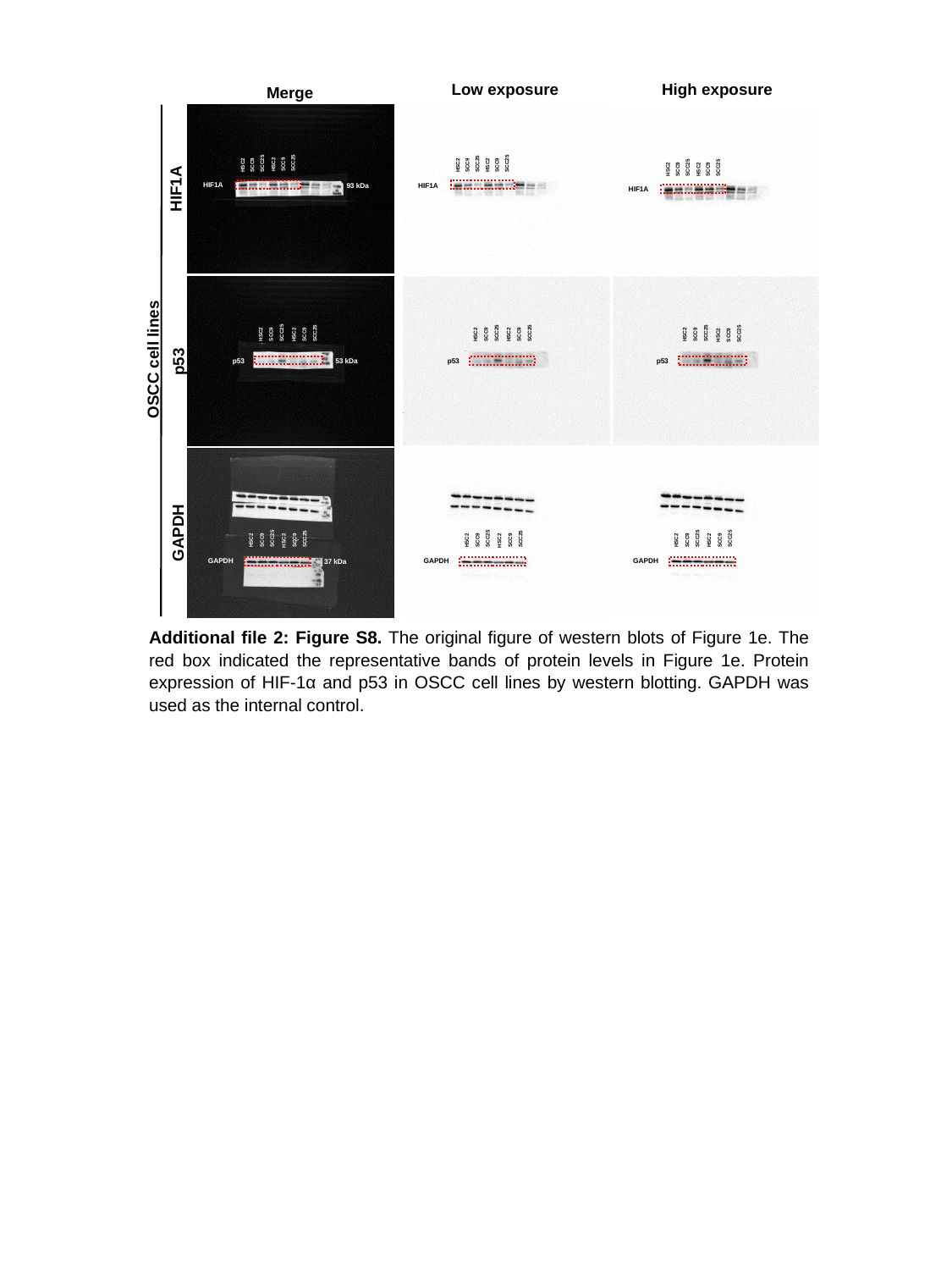

Low exposure
High exposure
Merge
SCC9
HSC2
SCC25
SCC9
HSC2
SCC25
SCC9
HSC2
SCC25
SCC9
HSC2
SCC25
SCC9
HSC2
SCC25
SCC9
HSC2
SCC25
HIF1A
HIF1A
HIF1A
HIF1A
SCC9
HSC2
SCC25
SCC9
SCC9
SCC9
SCC9
HSC2
SCC25
HSC2
HSC2
HSC2
SCC25
SCC25
SCC25
SCC9
HSC2
SCC25
OSCC cell lines
p53
p53
p53
p53
GAPDH
SCC9
SCC25
SCC9
SCC25
SCC9
SCC25
SCC9
SCC25
SCC9
SCC25
SCC9
SCC25
HSC2
HSC2
HSC2
HSC2
HSC2
HSC2
GAPDH
GAPDH
GAPDH
93 kDa
53 kDa
37 kDa
Additional file 2: Figure S8. The original figure of western blots of Figure 1e. The red box indicated the representative bands of protein levels in Figure 1e. Protein expression of HIF-1α and p53 in OSCC cell lines by western blotting. GAPDH was used as the internal control.

## Slide 3
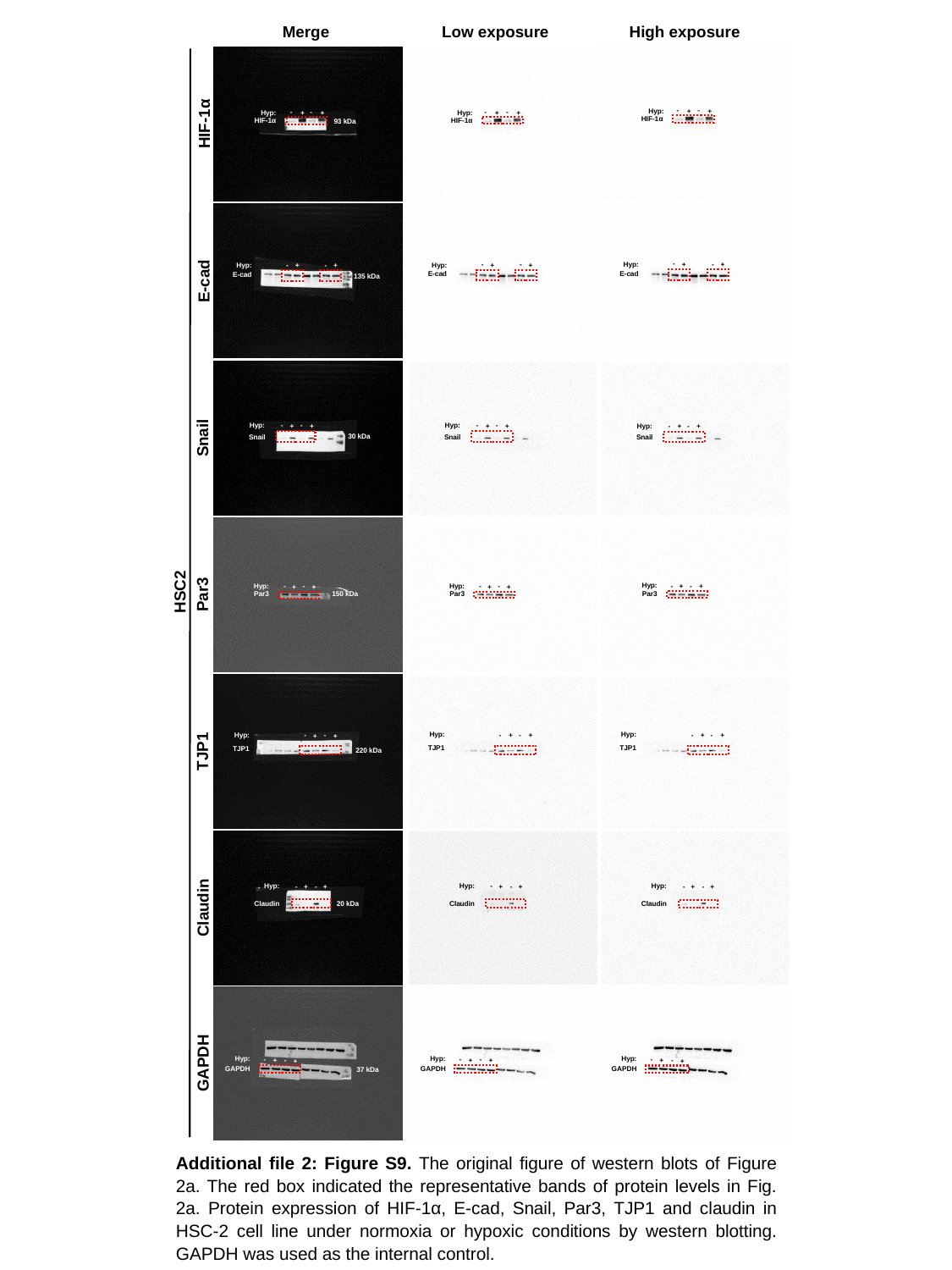

Merge
Low exposure
High exposure
-
-
+
+
Hyp:
-
-
-
-
+
+
Hyp:
+
Hyp:
+
HIF-1α
HIF-1α
HIF-1α
HIF-1α
-
-
+
-
-
Hyp:
+
+
+
-
Hyp:
-
+
Hyp:
+
E-cad
E-cad
E-cad
E-cad
-
Hyp:
-
Hyp:
-
-
Hyp:
-
-
+
+
+
+
+
+
Snail
Snail
Snail
Snail
Hyp:
-
Hyp:
-
Hyp:
-
+
+
-
-
-
+
+
HSC2
+
+
Par3
Par3
Par3
Par3
Hyp:
Hyp:
Hyp:
-
-
-
-
-
-
+
+
+
+
+
+
TJP1
TJP1
TJP1
TJP1
Hyp:
Hyp:
Hyp:
-
-
-
-
-
-
+
+
+
+
+
+
Claudin
Claudin
Claudin
Claudin
GAPDH
Hyp:
Hyp:
Hyp:
-
-
-
-
-
+
-
+
+
+
+
+
GAPDH
GAPDH
GAPDH
93 kDa
135 kDa
30 kDa
150 kDa
220 kDa
20 kDa
37 kDa
Additional file 2: Figure S9. The original figure of western blots of Figure 2a. The red box indicated the representative bands of protein levels in Fig. 2a. Protein expression of HIF-1α, E-cad, Snail, Par3, TJP1 and claudin in HSC-2 cell line under normoxia or hypoxic conditions by western blotting. GAPDH was used as the internal control.

## Slide 4
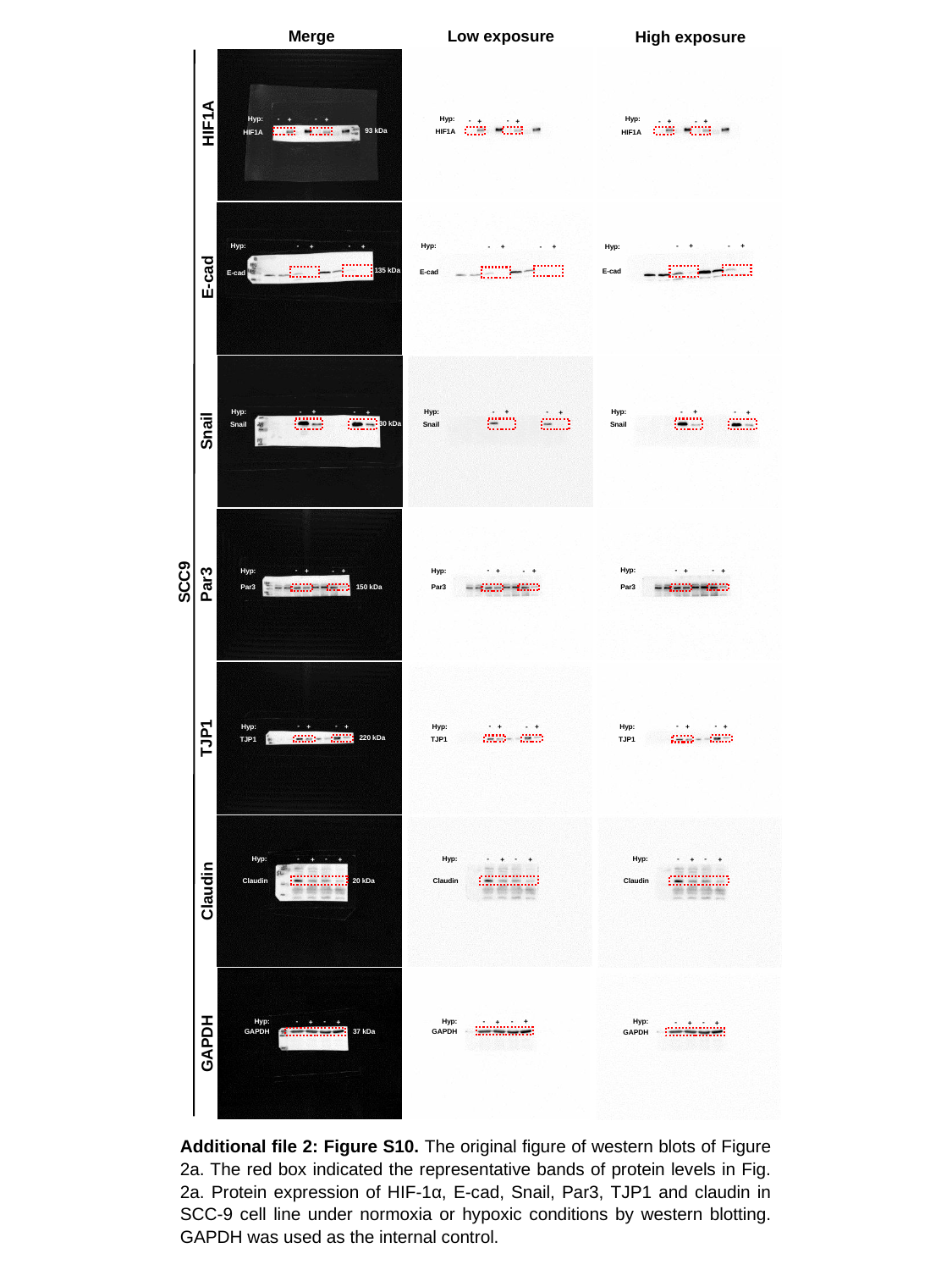

Merge
Low exposure
High exposure
HIF1A
-
Hyp:
Hyp:
-
Hyp:
+
+
-
-
-
+
-
+
+
+
93 kDa
HIF1A
HIF1A
HIF1A
-
-
-
-
Hyp:
Hyp:
+
+
+
+
Hyp:
-
-
+
+
135 kDa
E-cad
E-cad
E-cad
E-cad
Hyp:
-
Hyp:
-
-
Hyp:
-
-
-
+
+
+
+
+
+
30 kDa
Snail
Snail
Snail
Snail
-
-
Hyp:
-
-
-
-
Hyp:
Hyp:
+
+
+
+
+
+
SCC9
Par3
150 kDa
Par3
Par3
Par3
-
-
-
-
-
-
+
Hyp:
Hyp:
+
Hyp:
+
+
+
+
TJP1
220 kDa
TJP1
TJP1
TJP1
Hyp:
Hyp:
Hyp:
-
-
-
-
-
-
+
+
+
+
+
+
20 kDa
Claudin
Claudin
Claudin
Claudin
Hyp:
Hyp:
-
-
-
-
+
Hyp:
+
+
+
-
-
+
+
37 kDa
GAPDH
GAPDH
GAPDH
GAPDH
Additional file 2: Figure S10. The original figure of western blots of Figure 2a. The red box indicated the representative bands of protein levels in Fig. 2a. Protein expression of HIF-1α, E-cad, Snail, Par3, TJP1 and claudin in SCC-9 cell line under normoxia or hypoxic conditions by western blotting. GAPDH was used as the internal control.

## Slide 5
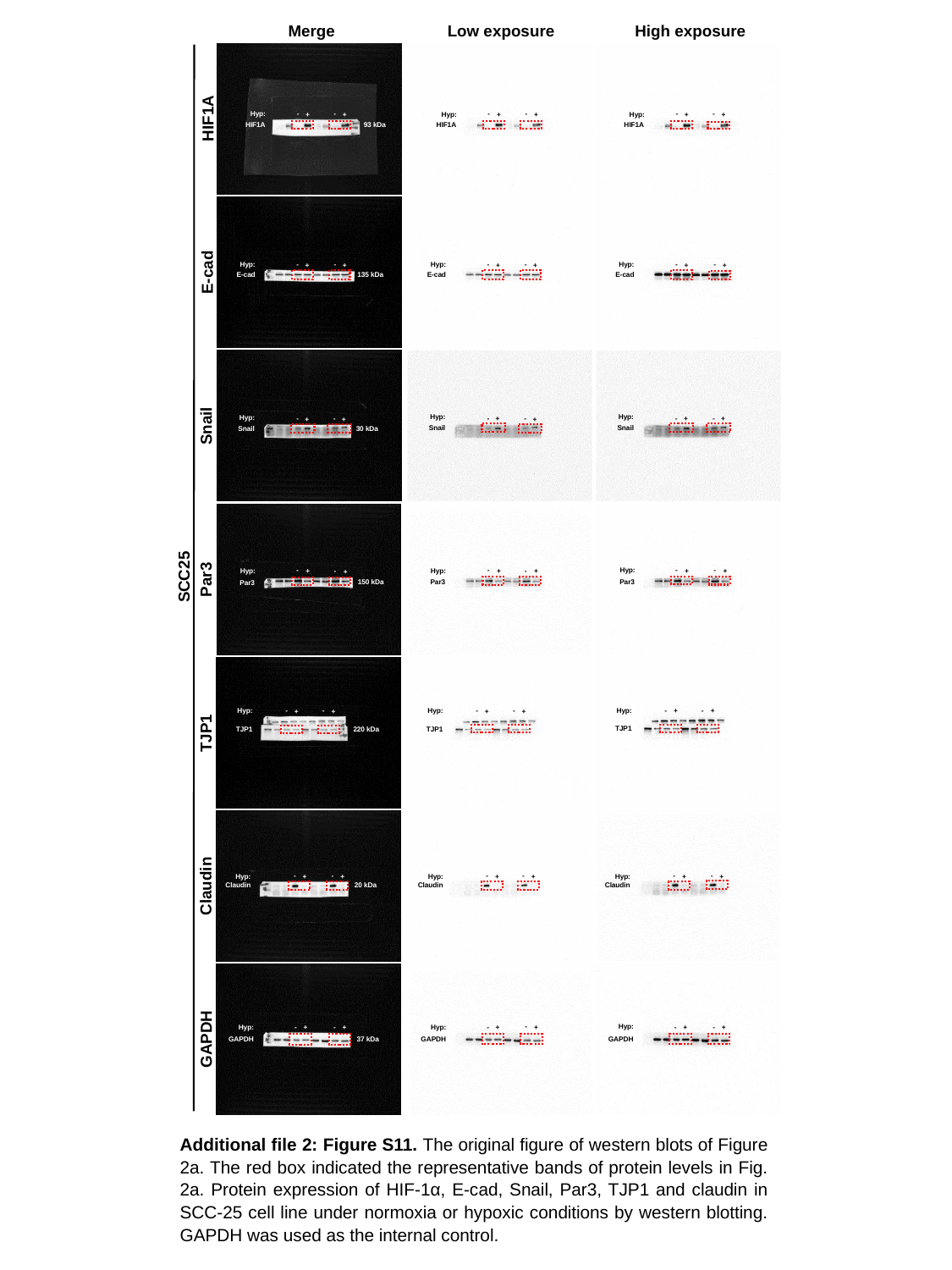

Merge
Low exposure
High exposure
HIF1A
-
-
-
-
-
Hyp:
-
Hyp:
Hyp:
+
+
+
+
+
+
HIF1A
HIF1A
HIF1A
-
-
-
-
-
Hyp:
Hyp:
Hyp:
-
+
+
+
+
+
+
E-cad
E-cad
E-cad
E-cad
Hyp:
Hyp:
Hyp:
-
-
-
-
-
-
+
+
+
+
+
+
Snail
Snail
Snail
Snail
-
-
Hyp:
-
-
+
-
Hyp:
-
Hyp:
+
+
+
+
+
SCC25
Par3
Par3
Par3
Par3
Hyp:
-
-
+
Hyp:
+
-
-
Hyp:
-
-
+
+
+
+
TJP1
TJP1
TJP1
TJP1
-
-
-
-
-
-
Hyp:
Hyp:
+
Hyp:
+
+
+
+
+
Claudin
Claudin
Claudin
Claudin
-
Hyp:
-
-
Hyp:
-
Hyp:
-
+
-
+
+
+
+
+
GAPDH
GAPDH
GAPDH
GAPDH
93 kDa
135 kDa
30 kDa
150 kDa
220 kDa
20 kDa
37 kDa
Additional file 2: Figure S11. The original figure of western blots of Figure 2a. The red box indicated the representative bands of protein levels in Fig. 2a. Protein expression of HIF-1α, E-cad, Snail, Par3, TJP1 and claudin in SCC-25 cell line under normoxia or hypoxic conditions by western blotting. GAPDH was used as the internal control.

## Slide 6
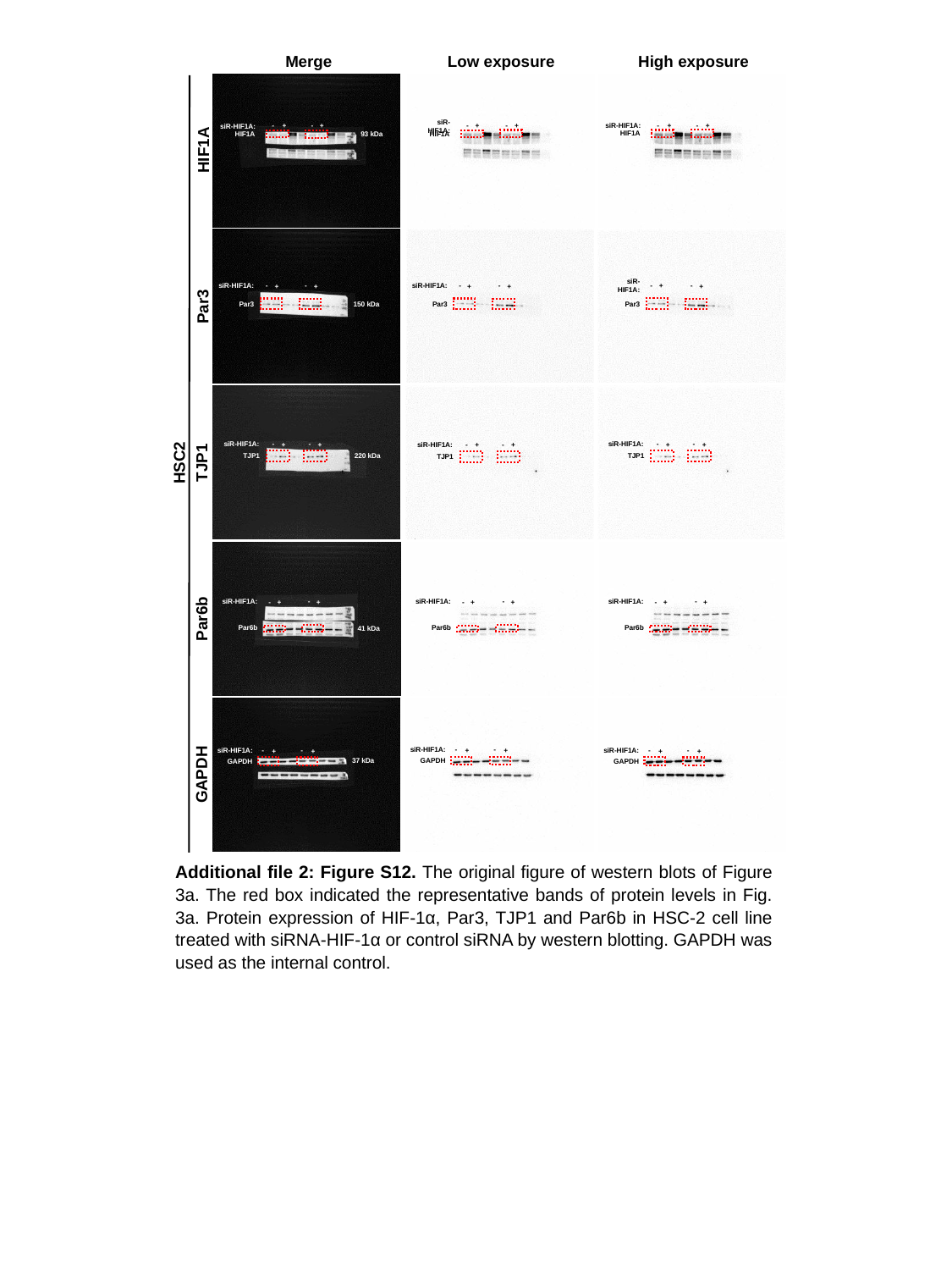

Merge
Low exposure
High exposure
-
-
-
-
-
-
+
+
+
+
siR-HIF1A:
+
+
siR-HIF1A:
siR-HIF1A:
HIF1A
HIF1A
HIF1A
HIF1A
-
-
-
-
siR-HIF1A:
-
-
siR-HIF1A:
siR-HIF1A:
+
+
+
+
+
+
Par3
Par3
Par3
Par3
-
-
-
siR-HIF1A:
-
siR-HIF1A:
+
+
+
-
+
-
siR-HIF1A:
+
+
TJP1
TJP1
TJP1
HSC2
TJP1
siR-HIF1A:
siR-HIF1A:
siR-HIF1A:
-
-
-
-
-
-
+
+
+
+
+
+
Par6b
Par6b
Par6b
Par6b
siR-HIF1A:
-
-
siR-HIF1A:
-
siR-HIF1A:
-
+
-
-
+
+
+
+
+
GAPDH
GAPDH
GAPDH
GAPDH
93 kDa
150 kDa
220 kDa
41 kDa
37 kDa
Additional file 2: Figure S12. The original figure of western blots of Figure 3a. The red box indicated the representative bands of protein levels in Fig. 3a. Protein expression of HIF-1α, Par3, TJP1 and Par6b in HSC-2 cell line treated with siRNA-HIF-1α or control siRNA by western blotting. GAPDH was used as the internal control.

## Slide 7
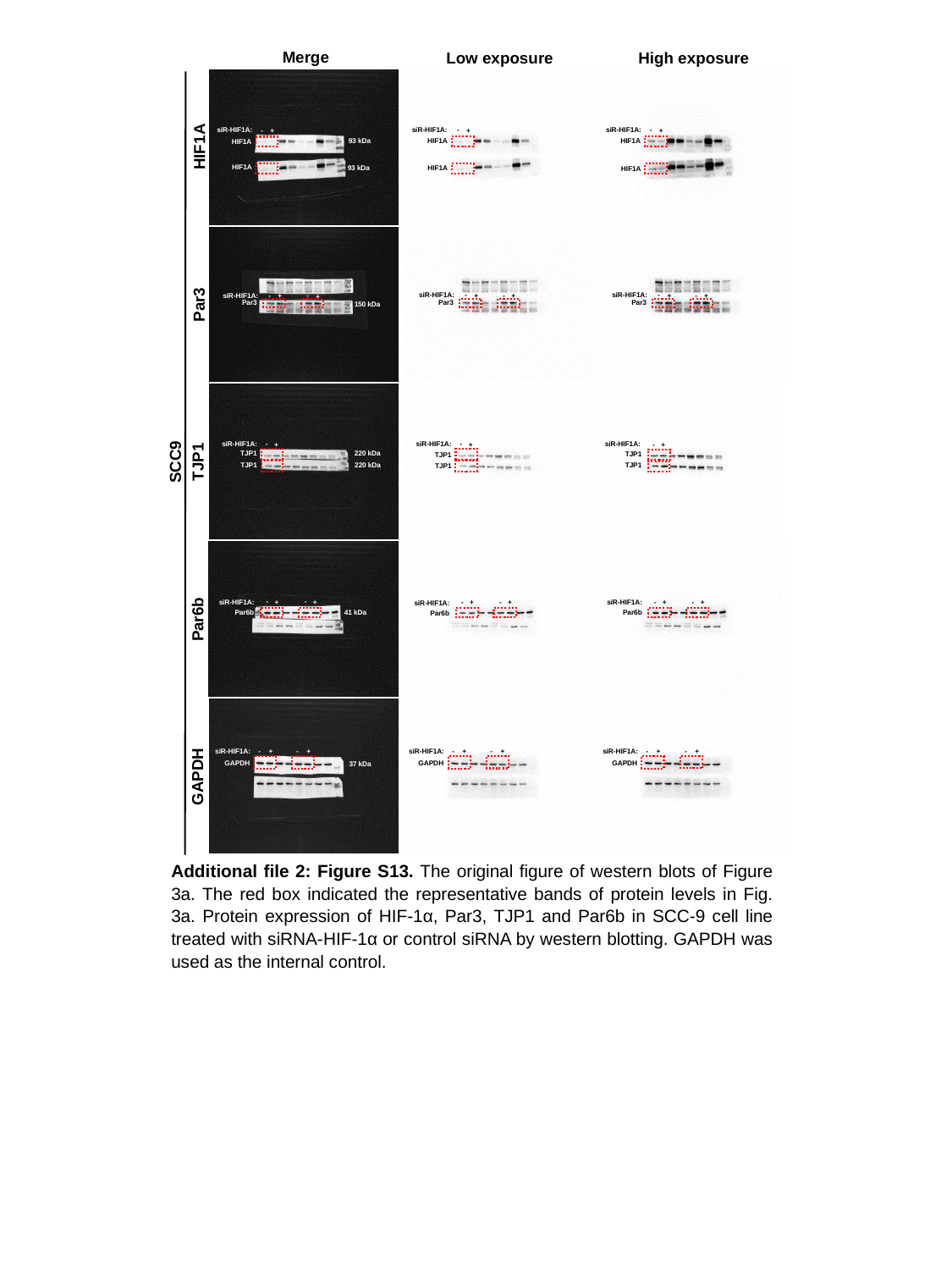

Merge
Low exposure
High exposure
siR-HIF1A:
-
siR-HIF1A:
siR-HIF1A:
-
-
+
+
+
93 kDa
HIF1A
HIF1A
HIF1A
HIF1A
HIF1A
HIF1A
93 kDa
HIF1A
siR-HIF1A:
-
siR-HIF1A:
-
siR-HIF1A:
-
-
-
+
-
+
+
+
+
+
Par3
Par3
Par3
Par3
150 kDa
siR-HIF1A:
siR-HIF1A:
-
-
siR-HIF1A:
-
+
+
+
TJP1
220 kDa
TJP1
TJP1
SCC9
TJP1
TJP1
220 kDa
TJP1
TJP1
-
-
-
-
-
-
+
+
+
+
siR-HIF1A:
+
siR-HIF1A:
+
siR-HIF1A:
41 kDa
Par6b
Par6b
Par6b
Par6b
-
-
siR-HIF1A:
-
siR-HIF1A:
-
siR-HIF1A:
-
-
+
+
+
+
+
+
GAPDH
GAPDH
GAPDH
37 kDa
GAPDH
Additional file 2: Figure S13. The original figure of western blots of Figure 3a. The red box indicated the representative bands of protein levels in Fig. 3a. Protein expression of HIF-1α, Par3, TJP1 and Par6b in SCC-9 cell line treated with siRNA-HIF-1α or control siRNA by western blotting. GAPDH was used as the internal control.

## Slide 8
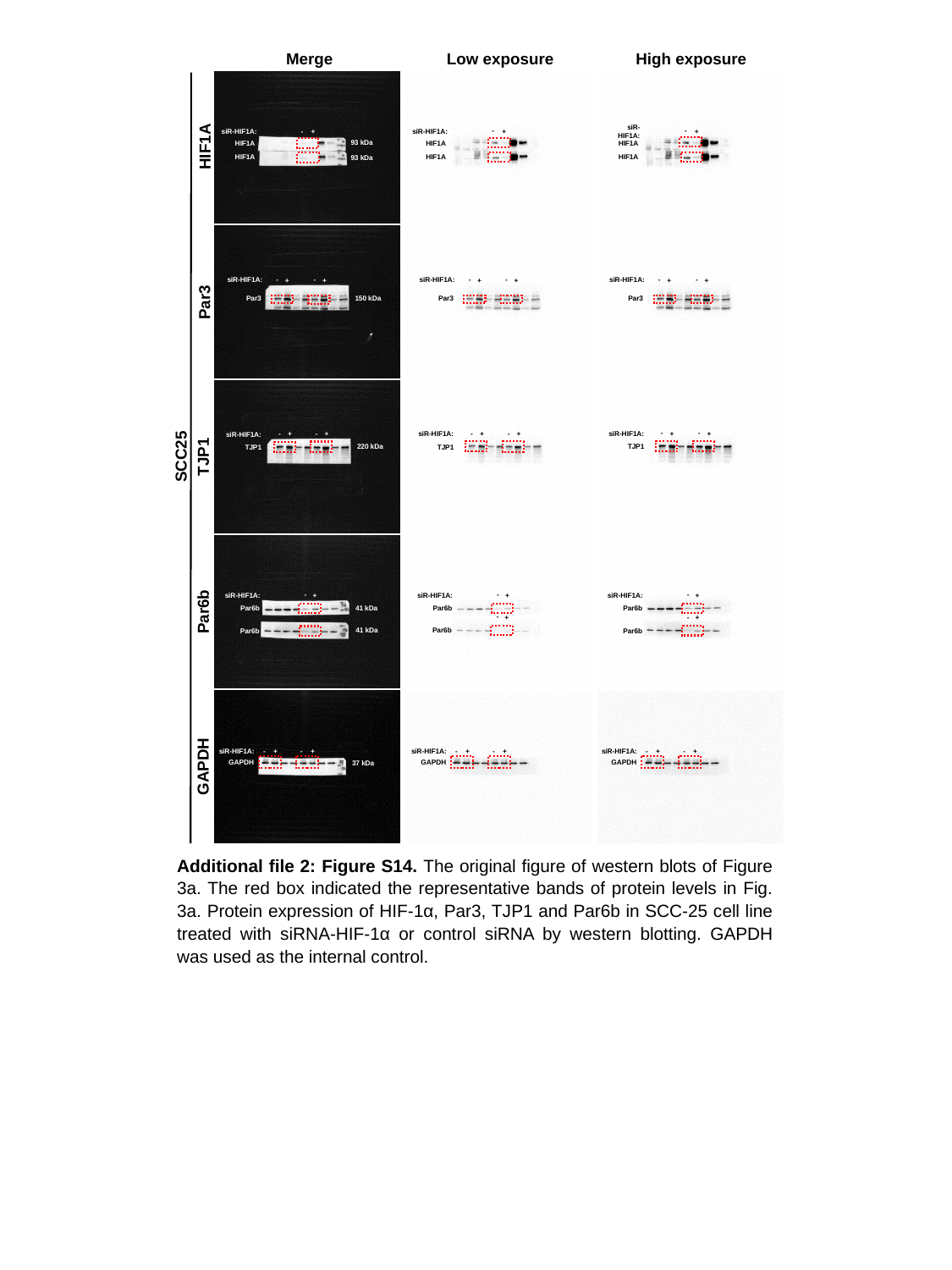

Merge
Low exposure
High exposure
-
-
+
+
siR-HIF1A:
siR-HIF1A:
-
+
siR-HIF1A:
HIF1A
HIF1A
HIF1A
HIF1A
HIF1A
HIF1A
HIF1A
-
-
-
-
-
-
siR-HIF1A:
siR-HIF1A:
siR-HIF1A:
+
+
+
+
+
+
Par3
Par3
Par3
Par3
-
-
-
-
-
+
-
+
siR-HIF1A:
+
+
+
+
siR-HIF1A:
siR-HIF1A:
TJP1
TJP1
TJP1
SCC25
TJP1
-
-
-
siR-HIF1A:
siR-HIF1A:
siR-HIF1A:
+
+
+
Par6b
Par6b
Par6b
Par6b
-
-
+
+
Par6b
Par6b
Par6b
-
-
siR-HIF1A:
siR-HIF1A:
-
-
-
siR-HIF1A:
-
+
+
+
+
+
+
GAPDH
GAPDH
GAPDH
GAPDH
93 kDa
93 kDa
150 kDa
220 kDa
41 kDa
41 kDa
37 kDa
Additional file 2: Figure S14. The original figure of western blots of Figure 3a. The red box indicated the representative bands of protein levels in Fig. 3a. Protein expression of HIF-1α, Par3, TJP1 and Par6b in SCC-25 cell line treated with siRNA-HIF-1α or control siRNA by western blotting. GAPDH was used as the internal control.

## Slide 9
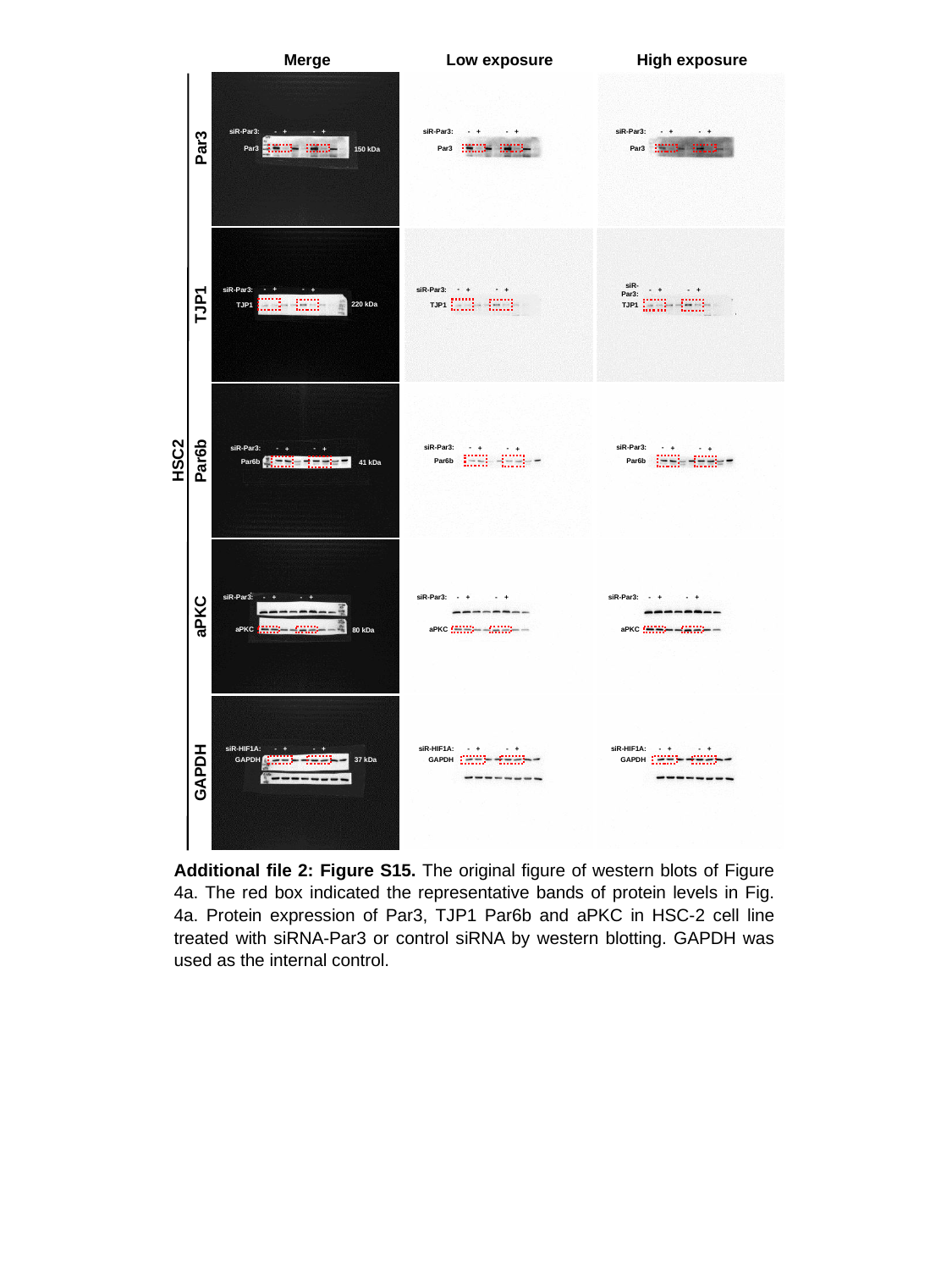

Merge
Low exposure
High exposure
-
-
-
-
-
-
+
+
+
+
+
siR-Par3:
siR-Par3:
siR-Par3:
+
Par3
Par3
Par3
Par3
150 kDa
-
-
+
-
-
-
-
+
+
+
siR-Par3:
siR-Par3:
+
+
siR-Par3:
TJP1
220 kDa
TJP1
TJP1
TJP1
siR-Par3:
-
siR-Par3:
-
-
siR-Par3:
-
-
+
+
-
+
+
+
+
HSC2
Par6b
Par6b
Par6b
Par6b
41 kDa
-
-
-
-
-
-
siR-Par3:
siR-Par3:
siR-Par3:
+
+
+
+
+
+
aPKC
aPKC
aPKC
aPKC
80 kDa
-
-
-
-
-
-
siR-HIF1A:
siR-HIF1A:
siR-HIF1A:
+
+
+
+
+
+
37 kDa
GAPDH
GAPDH
GAPDH
GAPDH
Additional file 2: Figure S15. The original figure of western blots of Figure 4a. The red box indicated the representative bands of protein levels in Fig. 4a. Protein expression of Par3, TJP1 Par6b and aPKC in HSC-2 cell line treated with siRNA-Par3 or control siRNA by western blotting. GAPDH was used as the internal control.

## Slide 10
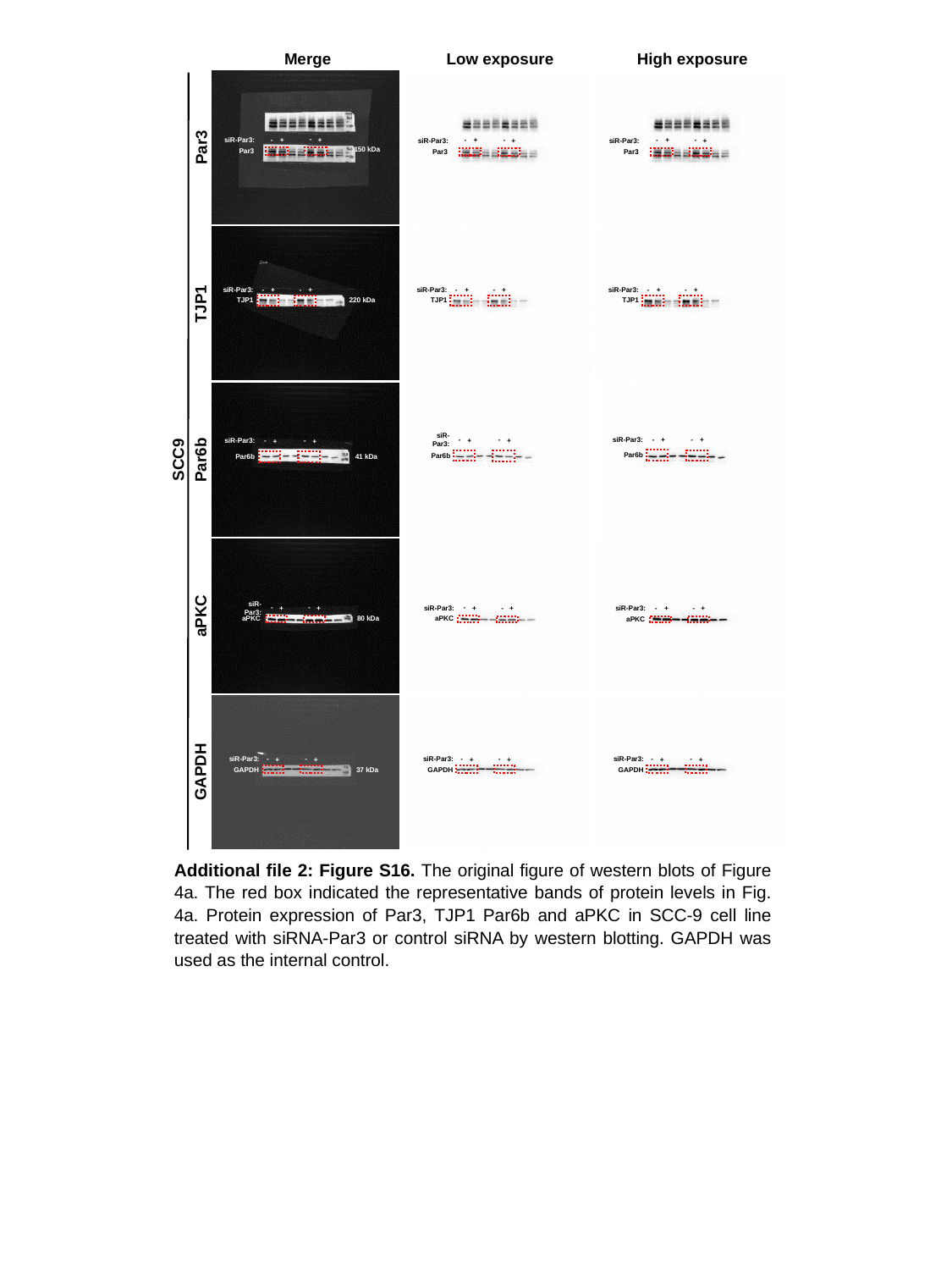

Merge
Low exposure
High exposure
-
-
-
-
+
-
-
+
siR-Par3:
+
+
+
+
siR-Par3:
siR-Par3:
Par3
150 kDa
Par3
Par3
Par3
-
-
-
-
-
-
+
+
+
siR-Par3:
siR-Par3:
siR-Par3:
+
+
+
TJP1
TJP1
220 kDa
TJP1
TJP1
siR-Par3:
-
-
siR-Par3:
-
+
-
+
+
+
siR-Par3:
-
-
+
+
SCC9
Par6b
Par6b
Par6b
Par6b
41 kDa
-
-
-
-
siR-Par3:
-
-
siR-Par3:
siR-Par3:
+
+
+
+
+
+
aPKC
aPKC
80 kDa
aPKC
aPKC
-
-
-
-
-
-
siR-Par3:
siR-Par3:
siR-Par3:
+
+
+
+
+
+
GAPDH
GAPDH
GAPDH
GAPDH
37 kDa
Additional file 2: Figure S16. The original figure of western blots of Figure 4a. The red box indicated the representative bands of protein levels in Fig. 4a. Protein expression of Par3, TJP1 Par6b and aPKC in SCC-9 cell line treated with siRNA-Par3 or control siRNA by western blotting. GAPDH was used as the internal control.

## Slide 11
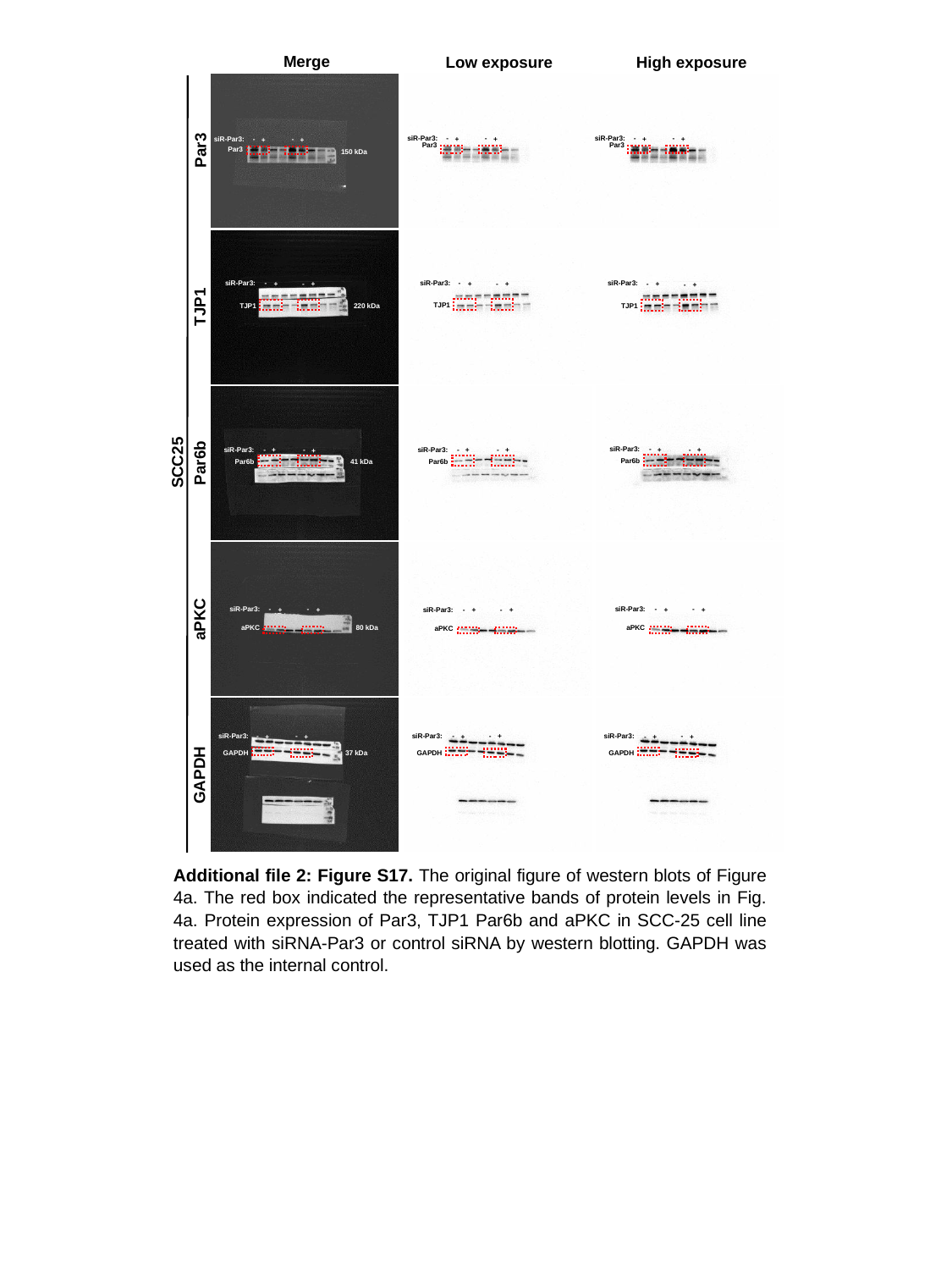

Merge
Low exposure
High exposure
-
-
-
siR-Par3:
-
siR-Par3:
+
+
+
+
-
-
siR-Par3:
+
+
Par3
Par3
Par3
Par3
150 kDa
siR-Par3:
-
-
siR-Par3:
siR-Par3:
+
-
+
-
-
+
+
+
-
+
TJP1
TJP1
TJP1
220 kDa
TJP1
-
siR-Par3:
-
-
siR-Par3:
+
-
-
+
siR-Par3:
-
+
+
+
+
SCC25
Par6b
Par6b
Par6b
41 kDa
Par6b
-
-
-
-
siR-Par3:
siR-Par3:
-
+
+
-
+
+
siR-Par3:
+
+
aPKC
80 kDa
aPKC
aPKC
aPKC
siR-Par3:
-
-
siR-Par3:
-
siR-Par3:
-
+
-
-
+
+
+
+
+
GAPDH
37 kDa
GAPDH
GAPDH
GAPDH
Additional file 2: Figure S17. The original figure of western blots of Figure 4a. The red box indicated the representative bands of protein levels in Fig. 4a. Protein expression of Par3, TJP1 Par6b and aPKC in SCC-25 cell line treated with siRNA-Par3 or control siRNA by western blotting. GAPDH was used as the internal control.
